# Zebrafish *WNK Lysine Deficient Protein Kinase 1 (wnk1)* Affects Angiogenesis Associated with VEGF Signaling

**DOI:** 10.1371/journal.pone.0106129

**Published:** 2014-08-29

**Authors:** Ju-Geng Lai, Su-Mei Tsai, Hsiao-Chen Tu, Wen-Chuan Chen, Fong-Ji Kou, Jeng-Wei Lu, Horng-Dar Wang, Chou-Long Huang, Chiou-Hwa Yuh

**Affiliations:** 1 Institute of Molecular and Genomic Medicine, National Health Research Institutes, Zhunan Town, Miaoli, Taiwan, ROC; 2 Institute of Biotechnology, National Tsing Hua University, Hsinchu, Taiwan, ROC; 3 Departments of Medicine, University of Texas Southwestern Medical Center, Dallas, Texas, United States of America; 4 College of Life Science and Institute of Bioinformatics and Structural Biology, National Tsing-Hua University, Hsinchu, Taiwan, ROC; 5 Department of Biological Science and Technology, National Chiao Tung University, Hsinchu, Taiwan, ROC; 6 College of Medicine, Kaohsiung Medical University, Kaohsiung, Taiwan, ROC; Medical College of Wisconsin, United States of America

## Abstract

The WNK1 (WNK lysine deficient protein kinase 1) protein is a serine/threonine protein kinase with emerging roles in cancer. WNK1 causes hypertension and hyperkalemia when overexpressed and cardiovascular defects when ablated in mice. In this study, the role of Wnk1 in angiogenesis was explored using the zebrafish model. There are two zebrafish *wnk1* isoforms, *wnk1a* and *wnk1b*, and both contain all the functional domains found in the human WNK1 protein. Both isoforms are expressed in the embryo at the initiation of angiogenesis and in the posterior cardinal vein (PCV), similar to fms-related tyrosine kinase 4 (*flt4*). Using morpholino antisense oligonucleotides against *wnk1a* and *wnk1b*, we observed that *wnk1* morphants have defects in angiogenesis in the head and trunk, similar to *flk1*/*vegfr2* morphants. Furthermore, both *wnk1a* and *wnk1b* mRNA can partially rescue the defects in vascular formation caused by *flk1*/*vegfr2* knockdown. Mutation of the kinase domain or the Akt/PI3K phosphorylation site within *wnk1* destroys this rescue capability. The rescue experiments provide evidence that *wnk1* is a downstream target for Vegfr2 (vascular endothelial growth factor receptor-2) and Akt/PI3K signaling and thereby affects angiogenesis in zebrafish embryos. Furthermore, we found that knockdown of vascular endothelial growth factor receptor-2 (*flk1*/*vegfr2*) or vascular endothelial growth factor receptor-3 (*flt4/vegfr3*) results in a decrease in *wnk1a* expression, as assessed by *in* *situ* hybridization and q-RT-PCR analysis. Thus, the Vegf/Vegfr signaling pathway controls angiogenesis in zebrafish via Akt kinase-mediated phosphorylation and activation of Wnk1 as well as transcriptional regulation of *wnk1* expression.

## Introduction

The development of the vascular system occurs via vasculogenesis and angiogenesis. Vasculogenesis refers to the de novo formation of vessels [1]; in angiogenesis, new blood vessels form by remodeling and extending old ones [2]. The most important molecules governing angiogenesis are the VEGF (vascular endothelial growth factor) family members and their receptors [3]. There are three different VEGF receptors, VEGFR1 (FLT1), VEGFR2 (KDR/FLK1) and VEGFR3 (FLT4). VEGFR2 mediates the majority of the downstream angiogenic effects of VEGF. These angiogenic effects include changes in microvascular permeability and endothelial cell proliferation, invasion, migration and survival. Upon activation by the binding of VEGF, the VEGFR2 tyrosine kinase phosphorylates downstream kinases, such as the phosphoinositide-dependent protein kinase (PI3 kinase), which then phosphorylates and activates the protein kinase Akt/PKB1. Multiple Akt/PKB substrates have been discovered, and WNK1 is a novel Akt/PKB substrate in insulin-stimulated 3T3-L1 adipocytes [4].

WNK1 (WNK lysine deficient protein kinase 1) protein is a novel mammalian serine/threonine protein kinase that lacks the invariant catalytic lysine found in subdomain II of all MAPKs, which is crucial for binding ATP and instead contains a catalytic lysine at position 233 in subdomain I [5]. Wnk1 cDNA was identified in a screen for MAPK family members in the mouse brain [5]. *WNK1* was found to be overexpressed in invasive colorectal cell lines [6]. WNK1 activates ERK5 by phosphorylating MEKK2/3, which is upstream of ERK5 in human embryonic kidney 293 (HEK293) cells [7]. Knocking down Wnk1 in the C17.2 mouse neural progenitor cell line resulted in decreased Erk5 activity, which reduced cell proliferation, migration and differentiation [8]. Other reports indicate that the WNK1 protein has a protein kinase AKT/protein kinase B (PKB) phosphorylation consensus sequence, and it has been shown that AKT kinase phosphorylates threonine 60 (Thr60) of WNK1 [9]. It is possible that during angiogenesis, VEGF/VEGFR2 phosphorylates and activates PI3 kinase, which then phosphorylates and activates AKT kinase, which might then phosphorylate WNK1.

WNK kinases contain an autophosphorylation domain, and serine 382 in the activation loop was shown to be required for autophosphorylation. The autoinhibitory domain, which is conserved in all four WNKs, suppresses the activity of the WNK1 kinase domain, and the two key residues required for the function of the autoinhibitory domain have been identified [10].

Overexpression of WNK1 causes hypertension and hyperkalemia in humans by altering renal Na^+^ and K^+^ transport [11]. WNK1 activates the downstream protein kinases STE20/SPS1-related proline-alanine-rich protein kinase (SPAK) and oxidative stress responsive 1 (OSR1) through phosphorylation of the t-loop in the catalytic domain [12], [13], [14], [15]. Activated SPAK and OSR1 associate and then phosphorylate and activate other ion co-transporters, including Na^+^/K^+^/2Cl^−^ co-transporter 1 (NKCC1) [16], [17]. NKCC1 is a ubiquitous ion transporter [18] that controls cell volume and maintains osmostasis through absorption of Na^+^, K^+^ and Cl^−^ ions [19], [20], suggesting that under hyperosmotic conditions, WNK1 can regulate the activity of NKCC1 through SPAK and OSR1 [21].

In the past, all research on WNK1 has focused on its function in cancer cell proliferation, differentiation, migration and apoptosis. In studies of somatic cells, WNK1 involvement in renal Na^+^ and K^+^ transport is also well known. However, the physiological function of WNK1 outside the kidney remains unclear. Using gene disruption and rescue in mice, Xie et al. found that Wnk1 function is required for embryonic angiogenesis and cardiac development, with *Wnk1* deletion affecting artery-vein specification [22]. The mechanism by which Wnk1 affects angiogenesis, however, remains largely unknown.

The zebrafish has emerged as a powerful vertebrate model system for development [23], [24], organogenesis [25], [26], [27], vasculogenesis [1], neurogenesis [28], [29] and carcinogenesis [30], [31]. Zebrafish and other vertebrates have highly conserved genomic sequences; thus, zebrafish can be used to analyze the developmental process of embryo formation as well as human disease pathology [1], [32]. Moreover, a range of forward and reverse genetic methods, including antisense morpholino oligonucleotide (MO)-based knockdown and Tol2 transgenesis, have been developed for functional analysis of genes in zebrafish [33], [34]. Green fluorescent protein (GFP) under the control of the *fli1* or *flk1* regulatory region is specifically expressed in angioblasts [35], [36]. The existence of transgenic lines expressing vessel-specific GFP also facilitates the study of vasculogenesis and angiogenesis in zebrafish.

Previous studies have shown that Wnk1 homozygous mutant mice die at E13 and have significant defects in angiogenesis, whereas Wnk1 heterozygous mice and wild-type mice do not differ significantly in hypotension, embryogenesis or angiogenesis [22], [37]. To further understand the role of Wnk1 in angiogenesis, we used the vessel-specific transgenic zebrafish line *Tg(fli1:EGFP)*. We found that knockdown of *wnk1* by MOs led to defects in the angiogenic sprouting of intersegmental vessels (ISVs). Unlike Wnk1 mutant mice, *wnk1* zebrafish morphants survive. Analysis of these morphants has furthered our understanding of the role Wnk1 plays during angiogenesis and helped to identify the signaling pathway through which Wnk1 functions.

## Materials and Methods

### Zebrafish husbandry

Zebrafish embryos, larvae, and adult fish were maintained in the Zebrafish Core Facility at NTHU-NHRI (ZeTH) according to established protocols and methods [38]. Animal use protocols for zebrafish were approved by the Institutional Animal Care Use Committee (IACUC) of the National Health Research Institutes. The animal protocol number is NHRI-IACUC-096037-A.

### Resources for sequence information

The zebrafish genomic sequence of WNK1 (NW_001878589) was obtained from the National Center for Biotechnology Information (NCBI). The human WNK1 protein sequence (gi|2711660|NP_061852.1) was obtained from the NCBI databank and used in a tBlastn search of the zebrafish RefSeq RNA database to identify zebrafish Wnk1 homologs.

### RNA extraction, RT-PCR and quantitative polymerase chain reaction (q-RT-PCR)

Total RNA was extracted from twenty embryos at different embryonic stages with a NucleoSpin RNA II kit (MACHEREY-NAGEL). Complement DNA was synthesized from 900 ng of RNA using MutiScribe Reverse Transcriptase with the following program: 25°C-10 min → 48°C-30 min → 95°C-5 min → 12°C-indefinite. After the RT reaction, we diluted the cDNA 20-fold and performed the q-RT-PCR as described below.

Quantitative PCR studies were performed on selected clones using gene-specific primers. The rate of PCR amplification in one population vs. another (after normalizing against housekeeping genes) reflects differences in the expression of the gene in the two populations. We used GFP DNA as our standard to calculate the number of molecules after a given cycle number. Each q-RT-PCR Ct (cycle number) can be converted into number of molecules according to the standard curve and then converted into molecules per embryo by dividing by the number of embryos used.

### DNA constructs: cloning and vectors

The *wnk1a* and *wnk1b* cDNA was generated by PCR amplification. The primers used to amplify the *wnk1a* and *wnk1b* cDNA are listed below.

*wnk1a* -F-*NotI*: 5′-ATAT**GCGGCCGC** ***ATG***GTCAAGTTCCTTTCCCC-3′ (bold letters indicate a *NotI* site, and *italic letters* denote the translation initiation site).

*wnk1a*-OUT-R: 5′-ACCCATTCGTGCCTCTATCA-3′.

*wnk1b*- F-NotI: 5′-ATAT**GCGGCCGC**CTGGAAAG***ATG***TCATCGGAAA-3′ (bold letters indicate a *NotI* site, and *italic letters* denote the translation initiation site).

*wnk1b*- OUT-R: 5′-CGTGGCATATTTGTGAGCAT-3′.

The PCR product was cloned into a cloning vector using the T&A cloning kit (Yeastern Biotech, catalog # YC001) and TaKaRa DNA ligation kit (TAKARA BIO INC, catalog #6024).

Rat Wnk1(1–449) was generated by PCR amplification from rat Wnk1(1–491)/pCMV5-Myc (kindly provided by Dr. Huang C. L., who obtained from Dr. Cobb’s lab). The primers used to amplify the rat Wnk1(1–449) are listed below.

Rat-*EcoRI*-Wnk1(1–449)-F: 5′-ATCG**GAATTC** *ATG*TCTGACGGCACCGCAGA-3′.(bold letters indicate an *EcoRI* site, and *italic letters* denote the translation initiation site).

Rat-*XbaI*-Wnk1(1–449)-R: 5′- ATCG**TCTAGA**AATTGCTACTTTGTCAAAAC-3′ (bold letters indicate an *XbaI* site).

Rat-Wnk1-seq-F: 5′-CCTAGTGTACCCGCAGTGGT-3′.

Rat-Wnk1-seq-R1: 5′-ACCACTGCGGGTACACTAGG-3′.

Rat-Wnk1-seq-R2: 5′-TAGCCATCTCAAGCATGCAC-3′.

The PCR product was digested with *EcoRI* and *XbaI* (NEW ENGLAND Biolabs Inc., catalog #R0101L and #R0145L), purified with a MinElute PCR Purification Kit (QIAGEN, catalog #28006), and subsequently cloned into an *EcoRI-* and *XbaI*-linearized pCS2+ vector using the T&A cloning kit (Yeastern Biotech, catalog # YC001) and TaKaRa DNA ligation kit (TAKARA BIO INC, catalog #6024).

### Microinjection

Microinjections were carried out as previously described [38]. For the MO experiments, we injected 2.5–15 ng of MO were microinjected into each one-cell-stage embryo. For the RNA injections, 150–600 pg of mRNA was microinjected into each one cell-stage embryo.

### Site-directed mutagenesis

We utilized the QuikChange II Site-Directed Mutagenesis Kit from Stratagene to generate the kinase dead and Akt phosphorylation site mutations in *wnk1. wnk1* cDNA in the yT&A vector (50 ng) was used as the template DNA in each reaction. Following amplification (95°C for 5 minutes, 18 cycles of 95°C for 30 seconds, 70°C for 30 seconds, and 68°C for 7 minutes 30 seconds), the product was treated with 1 µl of the *Dpn*I (10 U/µl) restriction enzyme to digest the parental methylated and hemimethylated dsDNA. The nicked vector DNA containing the desired mutations was then transformed into XL-1Blue super competent cells for nick repair and amplification.

The oligonucleotide sequences used to generate the mutations are given below. The underlined bases denote the mutation sites for site-directed mutagenesis.

Akt phosphorylation site mutant:

*wnk1a* 35 point mutation-F: 5′-CCGTCGTCGCCACCACGCCATGGATCGAGAACTGC-3′.

*wnk1a* 35 point mutation-R: 5′-GCAGTTCTCGATCCATGGCGTGGTGGCGACGACGG-3′.

Kinase-dead mutant:

*wnk1a* 206 point mutation-F: 5′-TAGGACGTGGCTCTTTTTGTACGGTCTACAAGGGACTG-3′.

*wnk1a* 206 point mutation-R: 5′-CAGTCCCTTGTAGACCGTACAAAAAGAGCCACGTCCTA-3′.

### Morpholinos used to knockdown gene expression

Morpholino oligonucleotides (MOs) were custom-made by Gene Tools (Philomath, OR, USA) and injected at doses that generated distinct gene knockdown effects while yielding the highest proportion of living embryos with the knockdown phenotype. The MO sequences and target sites are given below.

*wnk1a* ATG MO: 5′-ACTTGACCATCTTGTCGTTGAGATT-3′

(Against *wnk1a* translation start codon, –15 to +10.)

*wnk1a* 5MM MO: 5′-ACTTCACGATCTTCTCCTTGACATT-3′

(Against *wnk1a* translation start codon, –15 to +10, but containing the five underlined mismatches.)

*wnk1a* Up MO: 5′-TCCACCAAGTGGAGCGTGAAGTTAG-3′

(Against *wnk1a* upstream of translation start codon, –52 to –28.)

*wnk1b* Up MO: 5′-TGCGTAAATTTCCTGCTCTTGCTT-3′

(Against *wnk1b* upstream of translation start codon, –100 to –77.)

*wnk1b* ATG MO: 5′-TGGGATTTTCCGATGACATCTTTCC-3′

(Against *wnk1b* translation start codon, –6 to +19.)

*flk1* MO: 5′-GTCTGTTAAAATAACGTCCCGAATG-3′

(Against *flk1* upstream of translation start codon, –28 to –4.)

*flt4* MO: 5′-CTCTTCATTTCCAGGTTTCAAGTCC-3′

(Against *flt4* translation start codon, –17 to +8.)

*pi3kc2α*: 5′-TATGTGGGCCATGGTGTCAGCTCT-3′

(Against *pi3kc2α* translation start codon, –12 to +12.)

Lyophilized MOs were resuspended to a final concentration of 10 mM in sterilized ddH_2_O, and the solution was heated for 5 minutes at 65°C to dissociate aggregates of the powder. The MO solution was divided into 10-µl aliquots. Before use, each aliquot was heated to 65°C for 5 to 10 minutes and then cooled to room temperature.

### *In vitro* transcription of mRNA

We used the mMESSAGE mMACHINE T7 Ultra kit (Applied Biosystems, catalog # AM1345) for *in* *vitro* transcription of wild-type *wnk1a* and *wnk1b* and the two *wnk1a* mutants. The synthesized mRNAs were checked by agarose gel electrophoresis with RNA Millennium Markers (Applied Biosystems catalog # AM7150) for integrity. The concentration of *in* *vitro* transcribed mRNA was determined using a NanoDrop ND-1000 UV-Vis Spectrophotometer. Rat Wnk1(1–449) mRNA was generated from a pCS2+ vector construct by *in* *vitro* transcription using the mMESSAGE mMACHINE SP6 kit (Applied Biosystems, catalog # AM1340).

### Whole-mount *in* *situ* hybridization (WMISH)

Whole-mount *in* *situ* hybridization was carried out as previously described [38].

To generate probes for *in* *situ* hybridization, we first amplified *wnk1a*, *wnk1b*, *flt4*, *vegfc*, and *etv2* cDNA by PCR. The primers used to generate the DNA templates are listed in the supporting data (Data S1). We used the MEGAscript T7 kit (Ambion, catalog # AM1333) for *in* *vitro* transcription of digoxigenin (DIG)-labeled and fluorescein-labeled probes. Zebrafish embryos were collected and fixed in 4% paraformaldehyde at the indicated time points. To optimize hybridization, 5% dextran sulfate was added to the hybridization buffer for the DIG-labeled probes [39]. After post-hybridization washes to remove excess probe, the embryos were blocked with 1% blocking reagent (Roche Molecular Biochemicals, Mannheim, Germany) for 1 hour and then incubated overnight with pre-absorbed alkaline phosphatase (AP)-conjugated anti-DIG antibody (Roche Molecular Biochemical; 1∶2000 dilution) at 4 °C. Transcripts were visualized by AP-based NBT/BCIP staining under identical conditions and staining times.

To confirm the localization of *wnk1a* and *wnk1b* in the PCV of zebrafish embryo, double *in* *situ* hybridization was performed using fluorescein-labeled *wnk1a* or *wnk1b* probe mixed with DIG-labeled *flt4* probe and detected with an AP-conjugated anti-fluorescein antibody followed by an AP-conjugated anti-DIG antibody. The *wnk1a* and *wnk1b* transcripts were visualized by AP-based NBT/BCIP, and the *flt4* and *vegfc* transcripts were visualized by AP-based fast red (Roche Molecular Biochemicals, Mannheim, Germany) staining. Therefore, *wnk1a* and *wnk1b* expression appears blue while *flt4* expression is labeled in red. In between visualization of the fluorescein- and DIG-labeled probes, the AP enzymatic reaction was inactivated by incubating embryos in 100 mM glycine-hydrochloride (pH2.2) for 30 minutes.

### Construction of *wnk1a-GFP* and *wnk1b-GFP* for testing morpholino knockdown efficiency and specificity

To test the knockdown efficiency of *wnk1a* and *wnk1b* MOs, 263-bp and 286-bp fragments of *wnk1a* and *wnk1b* mRNA containing the MO targeting sites were amplified from zebrafish cDNA by PCR. For the *wnk1a-GFP* construct, the primers used were *wnk1a*-EcoRI (5′-AATA**GAATTC**TTCCACTTGGTTTAAAGCGG-3′, bold letters indicate an *EcoRI* site) and *wnk1a*-BamHI (5′-AATA**GGATCC**GCCTTCAGCAGTTCTCGATC-3′, bold letters indicate a *BamHI* site). For the *wnk1b-GFP* construct, the primers used were *wnk1b*-EcoRI (5′-AATA**GAATTC**AACTCTGTGGTTCACGTGAG-3′, bold letters indicate an *EcoRI* site) and *wnk1b*-BamHI (5′- AATA**GGATCC**TGGCGTCGCTTTCTGAC-3′, bold letters indicate a *BamHI* site). The PCR products were cloned into the pEGFP-N1 plasmid. Linearized *wnk1a-GFP* and *wnk1b-GFP* plasmids (200 pg) were microinjected into one-cell-stage embryos along with 10 ng of various *wnk1a* and *wnk1b* MOs as shown in Fig. S1.

### Fluorescent microscopy

Embryos were removed from their chorions with watchmaker forceps. For live imaging, the embryos were anesthetized with 0.168 mg/ml tricaine (Sigma, catalog # A-5040) and placed on a 1% agarose plate. For imaging of fixed tissues, the embryos were incubated in a 4% formaldehyde-PBS (1x PBS) solution overnight at 4°C, washed with PBST (1x PBS, 0.1% Tween 20) and then mounted in 1% PBS-low melt agarose (Zymeset). Embryos of different stages were collected for GFP visualization and photography. All imaging was performed on a Zeiss SteREO Discovery.V8 fluorescent microscope equipped with a Zeiss AxioCam MRc CCD camera.

## Results

### Identification of zebrafish *wnk1* and cloning of full-length *wnk1* cDNA

To identify the zebrafish *wnk1* orthologue, the human WNK1 protein sequence was used to search the translated zebrafish RefSeq RNA database using tBLASTn. Four sequences, XM_684564.5 (*wnk1*), XM_002666846.2 (*wnk1*), XM_003201205.1 (*wnk3*-like), and XM_680072.5 (*wnk4*-like), were found to be homologous to human WNK1.

We designed primers based on the first two homologs and cloned *wnk1* from zebrafish using RT-PCR. The zebrafish *wnk1a* cDNA is 4794 bp long, which is slightly different from the previously reported NCBI GenBank sequence XM_678215.3 and very similar to XM_002666846.2. The zebrafish *wnk1b* cDNA is 5730 bp long and similar to XM_684564.5 with large internal deletions.

**Figure 1:**
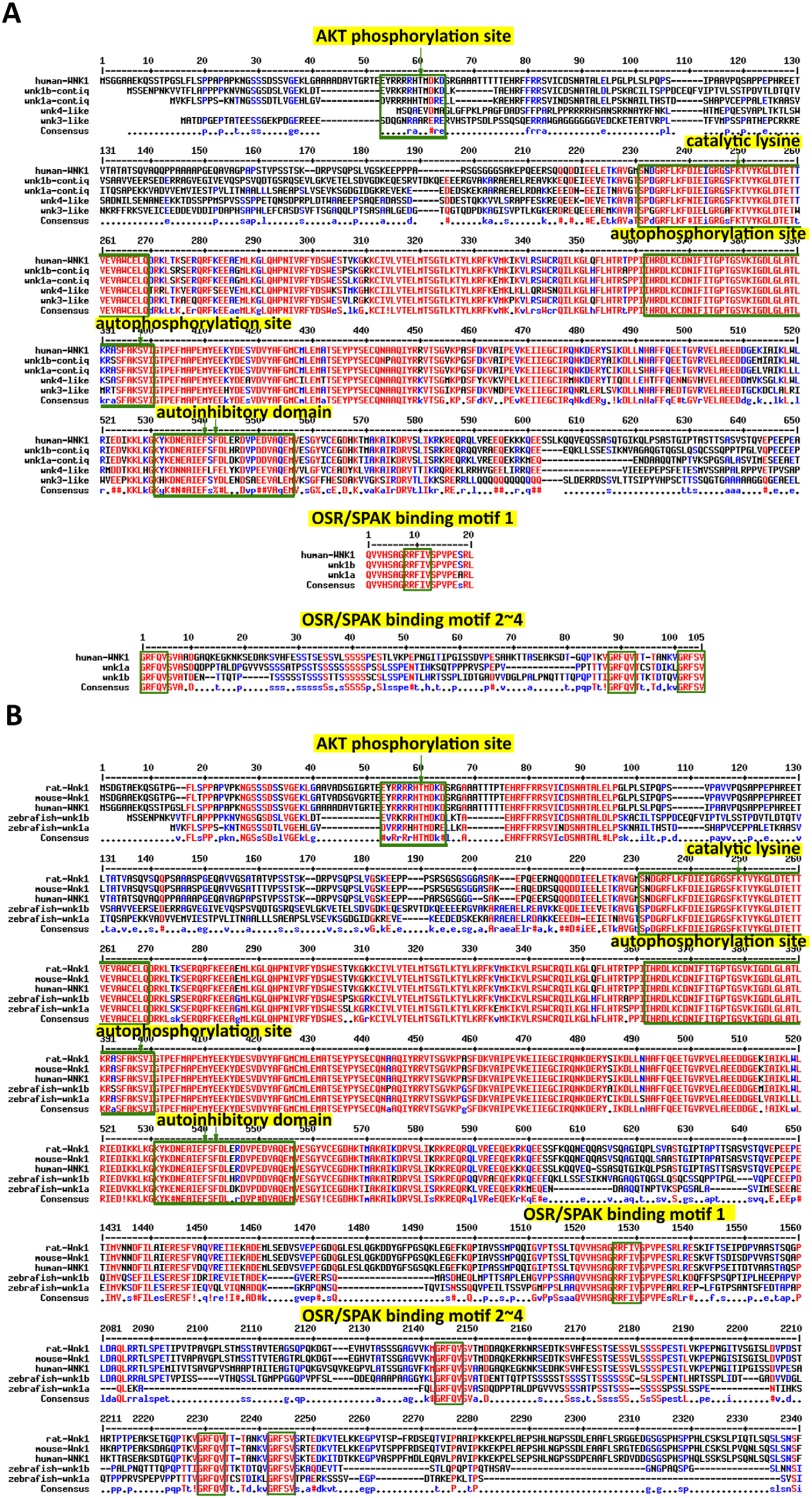
Sequence comparison between human WNK1 and zebrafish Wnk proteins. (**A**) Alignment of four zebrafish Wnks with the human WNK1 protein sequence. The WNK1 signature domain is highlighted. The AKT phosphorylation site at threonine 60 (arrow) in WNK1 is specific to Wnk1a and Wnk1b. The catalytic lysine (arrow) is located in a highly conserved region among all Wnk1s. The autophosphorylation site and the autoinhibitory domain are conserved between all four Wnk1s. The four OSR/SPAK binding motifs found in human WNK1 are specific to Wnk1a and Wnk1b. (**B**) Alignment of WNK1 protein sequences from mouse, rat, human and zebrafish.

The translated amino acid sequences of the four zebrafish *wnks* were compared with the human WNK1 protein sequence (Fig. 1A). As in human WNK1, a glycine in the glycine string of the MAPK motif is replaced by lysine in all four Wnk1 homologs in zebrafish. Additionally, all four zebrafish Wnk proteins contain an auto-inhibitory domain and a serine residue that is susceptible to auto-phosphorylation (Fig. 1A).

Among the four mammalian WNK family members, only WNK1 has an Akt phosphorylation site and the four RFXV (arginine-phenylalanine-any amino acid-valine) motifs required for binding to oxidative stress responsive 1 (OSR1) and STE20/SPS1-related proline-alanine-rich protein kinase (SPAK). Akt can phosphorylate WNK1, and OSR1 and SPAK are endogenous substrates for WNK1. Of the four zebrafish Wnks, only Wnk1a and Wnk1b have the Akt phosphorylation site and four OSR1/SPAK binding motifs (Fig. 1A). Based on these unique signatures, we confirmed that zebrafish *wnk1a* and *wnk1b* are mammalian *WNK1* homologs.

The WNK1 amino acid sequences from rat, mouse, human and zebrafish were also compared (Fig. 1B). In all examined species, the Wnk1 protein exhibited strong similarity in the AKT phosphorylation site, the catalytic domain, the autophosphorylation domain, the autoinhibitory domain and four OSR/SPAK binding domains. Previous work on Wnk1 has identified important residues for each of the domains [10]; these residues are also conserved between species. The rat Wnk1(1–449) truncation has been proven to be constitutively active using an *in* *vitro* kinase assay [10].

### Spatial and temporal expression of *wnk1* in zebrafish development

**Figure 2:**
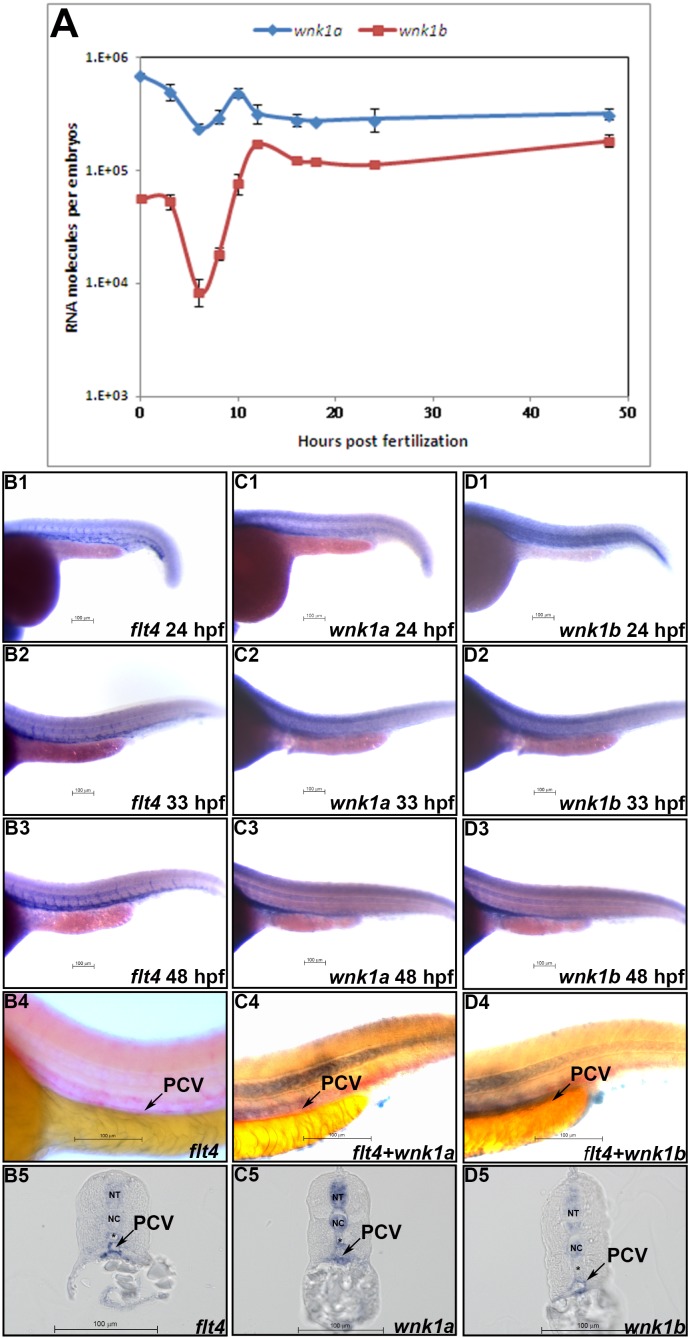
Spatial and temporal expression patterns of *wnk1a and wnk1b*. (A) *wnk1a* and *wnk1b* mRNA expression profiles as determined by q-RT-PCR. At least three replicates were performed, and the average number of molecules was calculated using a standard curve from a q-RT-PCR assay. The standard deviations are shown in the graph. The red and blue lines indicate the *wnk1a* and *wnk1b* expression profiles, respectively. (B∼D) Whole-mount *in* *situ* hybridization for *flt4* (B) *wnk1a* (C), and *wnk1b* (D) was performed at the indicated time points. *flt4* (B1∼B3), *wnk1a* (C1∼C3), and *wnk1b* (D1∼D3) mRNA expression in the tail is shown at 24, 33 and 48 hpf. Double *in* *situ* hybridization for *flt4* alone (B4), *wnk1a+flt4* (C4), and *wnk1b+flt4* (D4) shows the co-localization of *wnk1a* and *wnk1b* with *flt4* in the PCV. Expression of *flt4* (B5), *wnk1a* (C5) and *wnk1b* (D5) is seen in the PCV (arrow) in sections from stained embryos. *wnk1a* and *wnk1b* are also expressed in the neural tube (NT) and notochord (NC). The dorsal aorta (*) is negative for *wnk1a* and *wnk1b* expression. Scale bar: 100 µm.

To understand the role of *wnk1* in embryogenesis, we examined the temporal expression profile of *wnk1* by quantitative *reverse transcription polymerase chain reaction* (q-RT-PCR) (Fig. 2A). *wnk1a* and *wnk1b* mRNAs are maternally deposited, and zygotic expression of both begins as early as 10 hpf (hours post fertilization) and remains high at all stages examined. The expression of *flk1/vegfr2* begins as early as the 5 to 9 somite stage (12 to 15 hpf) in the intermediate cell mass of the mesoderm [40] and the 14 to 19 somite stage (16 to 19 hpf) in the head and vessel endothelial cells [41]. Additional studies have reported that *flk1/vegfr2* is expressed in the intersegmental vessels at 24 hpf [42] when the single circulatory loop in zebrafish is established [43], [44]. Overall, the temporal expression of *wnk1* is consistent with its role in angiogenesis. We did not detect significant expression of *wnk3* or *wnk4* in the zebrafish embryo prior to 48 hpf (data not shown).

q-RT-PCR was performed using cDNA from whole embryos, including non-vascular tissues. We next examined *wnk1a* and *wnk1b* expression in blood vessels by whole-mount *in* *situ* hybridization. At 3 hpf, maternal *wnk1a* and *wnk1b* mRNA are ubiquitously expressed in embryos (Fig. S2-A1, B1). At 6 and 8 hpf, expression of both *wnk1a* and *wnk1b* transcripts is decreased (Fig. S2-A2, A3, B2, B3). At 10 and 12 hpf, zygotic *wnk1a* and *wnk1b* are expressed ubiquitously (Fig. S2-A4, A5, B4, B5). At 18 hpf, *wnk1a* is strongly expressed in head and somite tissues (Fig. S2-A6), whereas *wnk1b* is also expressed in the notochord (Fig. S2-B6). Similarly, from 24 to 48 hpf, *wnk1a* is detected in the head, neural tube, somite, and PCV (Fig. S2-A7∼A12), whereas *wnk1b* is detected in all those tissues plus the notochord. Similar to *wnk1a*, *wnk1b* is expressed in the PCV after 33 hpf (Fig. S2-B7∼B12). We used *flt4,* which is known to be expressed in the posterior cardinal vein (PCV), as a marker and found that both *wnk1a* and *wnk1b* are expressed in the PCV from 24 to 48 hpf (Fig. 2A1∼A3, B1∼B3 and C1∼C3).

To confirm the localization of *wnk1a* and *wnk1b* in the PCV of zebrafish embryos, double *in* *situ* hybridization with antisense *wnk1a* or *wnklb* riboprobes and a *flt4* riboprobe was performed. The *flt4* transcript was detected with an AP-conjugated anti-DIG antibody and visualized with AP-Fast Red (Fig. 2A4) while *wnk1a* and *wnk1b* were detected with an AP-conjugated anti-fluorescein antibody. Our data showed that *wnk1a* and *wnk1b* colocalized with *flt4* in the PCV. We also observed *wnk1a* and *wnk1b* expression in the notochord (Fig. 2B4, C4). Embryos hybridized with the sense probes for *wnk1a* and *wnk1b* have no staining, indicating that the staining observed with the antisense probe is specific (Fig. S2-A13 and B13). Thus, colocalization studies using double *in* *situ* hybridization to detect both *wnk1a* or *wnk1b* and the PCV marker *flt4* in the same embryo indicate that *wnk1a* and *wnk1b* are expressed in vascular structures specific to the PCV.

We also examined sections of embryos following whole-mount *in* *situ* hybridization for *flt4*, *wnk1a* or *wnk1b*. We found *flt4* expression in the PCV whereas *wnk1a* and *wnk1b* expression was observed in the PCV, the neural tube (NT) and the notochord (NC) (Fig. 2A5, B5 and C5). All of these results suggest that *wnk1a* and *wnk1b* are indeed expressed in the vascular structures of the PCV.

### Quantitative analysis of the effect of *wnk1* knockdown on angiogenesis

To investigate the functional role of *wnk1* in zebrafish embryos, we used the *Tg(fli1:EGFP)* strain, which allows immediate and direct *in* *situ* monitoring of vessel formation. Translation of endogenous *wnk1* was suppressed in *Tg(fli1:EGFP)* by targeting *wnk1a* and *wnk1b* with specific antisense morpholino oligonucleotides (MOs). Morpholino specificity was verified by testing their ability to suppress the expression of a fusion protein that contained the targeted *wnk1* sequence (Fig. S1). The primer and nucleotide sequence for the *wnk1a-GFP* and *wnk1b-GFP* constructs are provided in the supporting data (Data S1). Figure S1 shows the target sites for the MOs, the number of injected embryos, and the quantification of GFP expression in the morphants. Morpholinos that target the 5′UTRs of *wnk1a* and *wnk1b* also efficiently inhibit the expression of the corresponding GFP fusion construct (Fig. S1-A4, B4). In contrast, the control MO does not suppress the expression of the GFP fusion protein (Fig. S1-A5, B5). Furthermore, *wnk1b* Up MOs do not inhibit *wnk1a-GFP* (Fig. S1-A6) and *wnk1a* MOs do not suppress *wnk1b-GFP* (Fig. S1-B6, B7). Our results demonstrate that *wnk1a* MOs specifically inhibit *wnk1a-GFP* expression and *wnk1b* MOs specifically inhibit *wnk1b-GFP* expression.

To determine the role that *wnk1* plays in angiogenesis, we designed and tested MOs against *wnk1a*, *wnk1b* and other genes required for angiogenesis. ISVs sprout and elongate dorsally from the DA and the PCV and connect to the DLAV between 29 and 36 hpf [45]. We analyzed embryos at 33 hpf, when the ISVs are forming. Because ISV development begins in the anterior trunk and proceeds posteriorly, we calculated the lengths of the ISVs from the middle of the yolk to the yolk extension. ISVs were observed in age-matched embryos and categorized as having extended over 100%, 75%, 50%, 25% or 0% of the distance from the DA (or PCV) to the DLAV (Fig. S3-A). Due to variations in the effects of morpholino injection from embryo to embryo, we examined many embryos in each group. The phenotype and motility of the embryos were classified as described in Fig. S3-B. The number of embryos analyzed (n) and the means, standard deviation and significance from three independent experiments are indicated in the figures.

**Figure 3:**
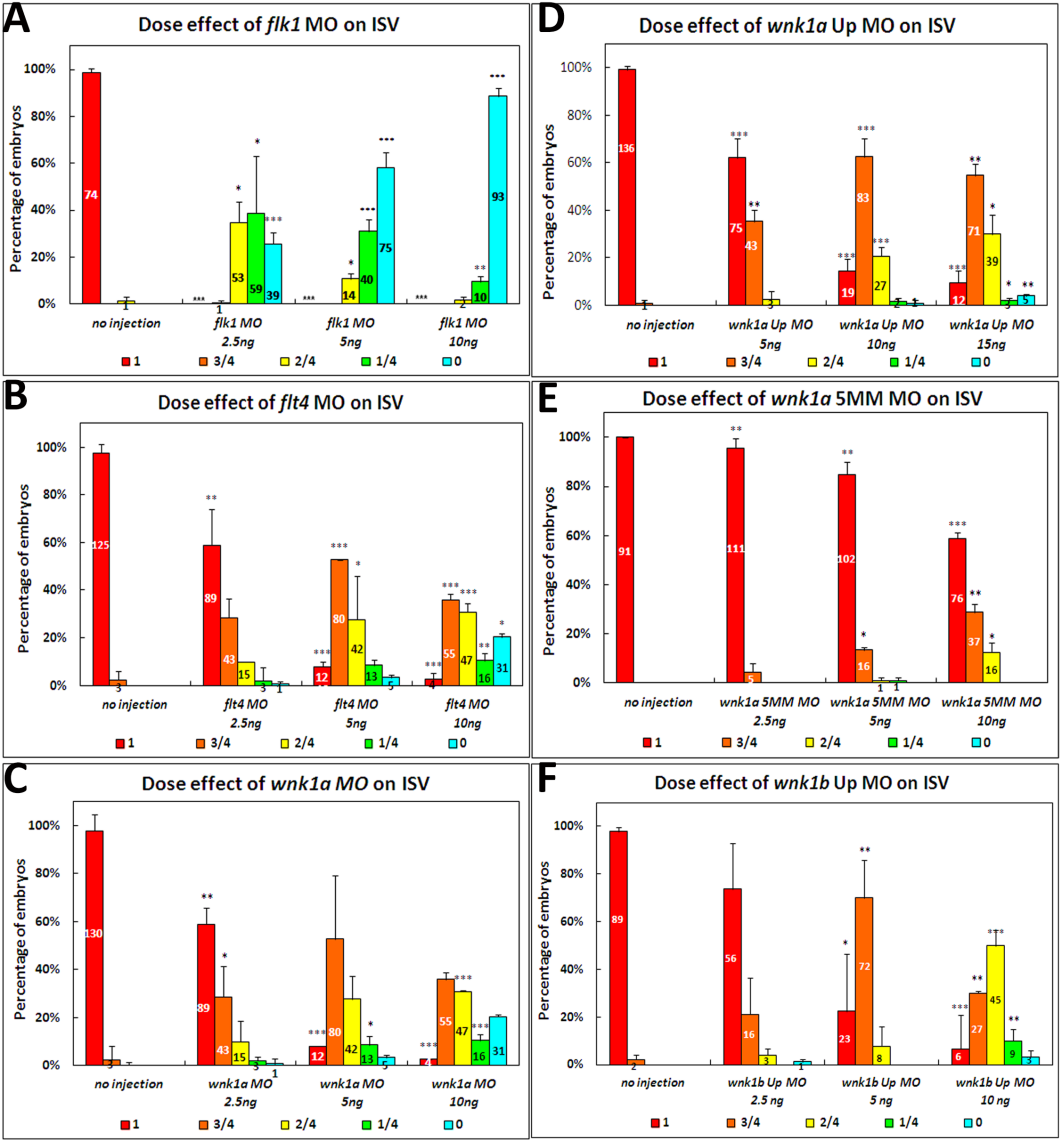
Statistical analysis of the length of intersegmental vessels in *flk1*, *flt4*, *wnk1a*, *wnk1a* 5MM, *wnk1a* upstream and *wnk1b* upstream morpholino-injected embryos. The effects of (A) *flk1* MO, (B) *flt4* MO, (C) *wnk1a* MO, (D) *wnk1a* 5 base mismatch MO, (E) *wnk1a* upstream MO, and (F) *wnk1b* upstream MO on the length of intersegmental vessels. Morpholino injection has a dose-dependent effect on intersegmental vessel formation and growth. All experiments were performed at least three times, and the average ISV length was calculated along with the standard deviation, which is labeled on each bar. Red indicates that the ISVs grew to full length, orange indicates that the ISVs were 75% of the normal length, yellow indicates 50%, green indicates 25%, and light blue indicates that no ISVs were observed in the embryos. The differences between treatments were assessed using a two-tailed Student’s *t*-test. Significant differences between the morphants and controls are indicated (_*_, *P*<0.05; _**_, *P*<0.01; and _***_, *P*<0.001).

In mice, VEGFR2 (KDR/FLK1) is the primary VEGFA receptor in the developing endothelial lineage and it is essential for endothelial differentiation during embryonic vasculogenesis as well as angiogenesis [46]. VEGFR2 also plays important roles in physiological as well as pathological postnatal angiogenesis [47]. In zebrafish, VEGFR2 is essential for angiogenesis only, and knockdown of *vegfr2/flk1* using morpholinos causes angiogenesis defects as shown by the shortening of ISVs (Fig. 3A). The inhibition of ISVs by *flk1* MO knockdown is dose-dependent.

VEGFR3 (Flt4) is predominantly expressed in embryonic blood endothelium, lymphatic endothelial cells, monocytes and macrophages [48]. VEGFR3 is important in lymphangiogenesis, but recent studies have shown that VEGFR3 also functions during sprouting angiogenesis [49]. In mouse, blocking VEGFR3 suppresses angiogenic sprouting and reduces the retina vessel density [50]. We found that *flt4* MOs inhibit the formation of ISVs, although to a lesser extent than *flk1* MOs (Fig. 3B).

Compared with *vegfr2* and *vegfr3* morphants, *wnk1a* morphants display a similar inhibition of ISV growth (Fig. 3C), suggesting that *wnk1a* is involved in angiogenesis in the vessel during sprouting or elongation. A 5-base mismatch (5MM) control morpholino has no inhibitory activity, with ISV lengths in the morphants comparable to those in wild-type embryos (Fig. 3D). A second morpholino targeted to the 5′-untranslated region of *wnk1a* causes similar inhibition (Fig. 3E). We also designed a translation-inhibiting MO and an upstream MO for *wnk1b* knockdown and found that the *wnk1b* upstream MO significantly inhibits ISV growth (Fig. 3F).

### Zebrafish *wnk1* knockdown results in defective angiogenesis but not vasculogenesis

**Figure 4:**
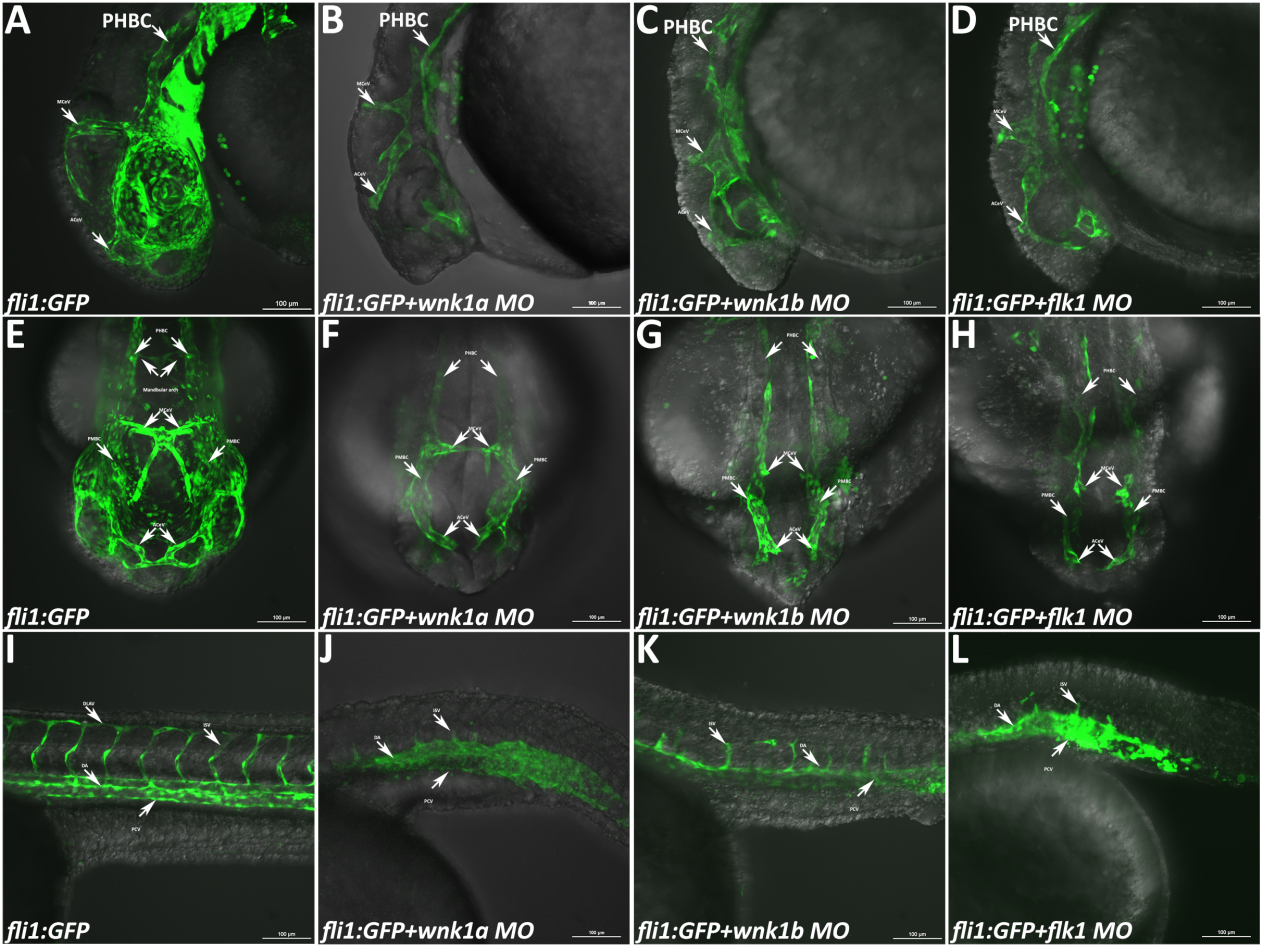
Phenotype of *Tg(fli1:GFP)* embryos injected with various morpholinos and imaged with a confocal microscope. (A–D) Lateral views of the heads of uninjected control embryos and *wnk1a*, *wnk1b* and *flk1* morphants at 33 hpf. (E–H) Frontal views of the heads of uninjected control embryos and *wnk1a*, *wnk1b* and *flk1* morphants at 33 hpf. (I–L) Lateral views of the trunk in uninjected control embryos and *wnk1a*, *wnk1b* and *flk1* morphants at 33 hpf. Important vessels are indicated with arrows and labeled, with the full name given in the text. Scale bar: 100 µm.

Representative age/somite matched images of uninjected *Tg(fl1i:EGFP)* embryos as well as *wnk1a*, *wnk1b*, and *flk1* morphants are shown in Figure 4. Since *wnk1a* and *wnk1b* are expressed strongly in the head as well as the trunk, we examined the embryos from a lateral head view (Fig. 4A–D), a frontal head view (Fig. 4E–H), and a lateral trunk view (Fig. 4I–L). Knockdown of either *wnk1a* or *wnk1b* causes significant defects in angiogenesis in both the head and trunk vessels. The vessel formation defects caused by knockdown of *wnk1a* or *wnk1b* in zebrafish are similar to the defects observed in *Wnk1* mutant mice, which include atresic branches of the internal carotid artery and primary head veins and disorganization of the intersomitic vessels and their branches [22]. Knockdown of either *wnk1a* or *wnk1b* in zebrafish inhibits the density and formation of vessels in the head region and body (Fig. 4). Notably, only the formation of small vessels in the head, such as the caudal division of the internal carotid artery (CaDI), the primitive mesencephalic artery (PMsA) and the partial optic artery (OA), are inhibited. Major vessel structures, such as the vessels of the mandibular arch, the anterior (rostral) cerebral vein (ACeV), the cranial division of the internal carotid artery (CrDI), the middle cerebral vein (MCeV), the primordial hindbrain channel (PHBC), the primitive internal carotid artery (PICA), and the primordial midbrain channel (PMBC), are not affected by *wnk1a* MO (Fig. 4B, F, J) or *wnk1b* MO (Fig. 4C, G, K); this result is similar to that seen in *flk1* morphants (Fig. 4D, H, L).

In the trunk, we found that growth of the intersegmental vessels (ISVs) is inhibited in *wnk1* morphants and that formation of the dorsal longitudinal anastomotic vessel (DLAV) is also affected. However, blood vessels formed by the vasculogenesis process, including the dorsal aorta (DA), the caudal artery (CA), the caudal vein (CV) and the posterior cardinal vein (PCV), are not affected (Fig. 4I–L). The phenotype of zebrafish *wnk1* morphants is consistent with that of *wnk1* knockout mice, with effects on angiogenesis but not vasculogenesis [22].

### Expression of the vasculogenesis marker *etv2* is unaffected in *wnk1* morphants

**Figure 5:**
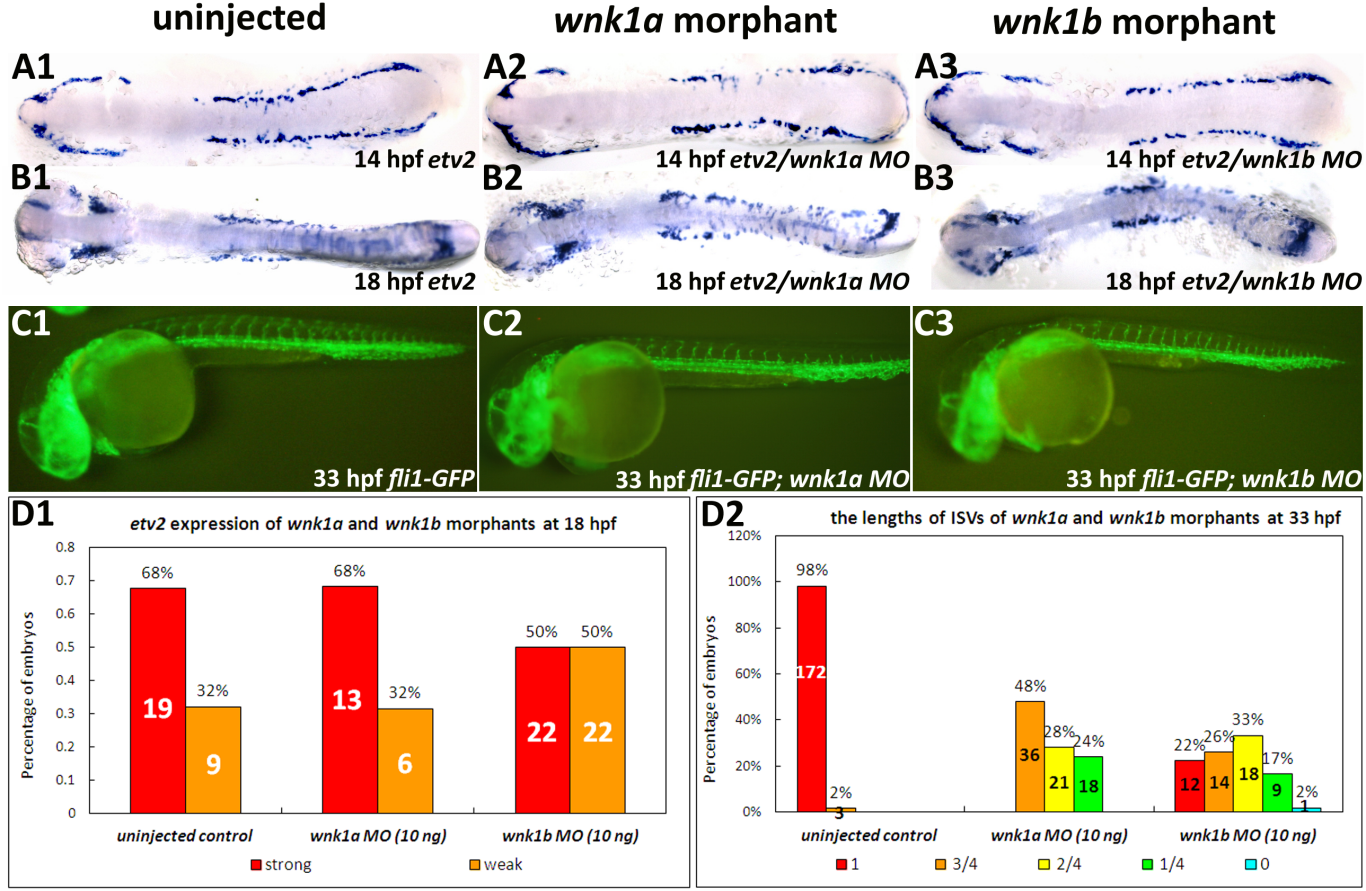
Effect of *wnk1a* or *wnk1b* knockdown on vasculogenesis and angiogenesis in *Tg(fli1:GFP)* embryos. (A, B) Whole mount *in* *situ* hybridization for *etv2* at 14 and 18 hpf in uninjected control embryos (A1, B1), *wnk1a* morphants (A2, B2), and *wnk1b* morphants (A3, B3). Flat mounts of de-yolked embryos were prepared. (C) GFP fluorescence was used to assay ISV formation in uninjected control embryos (C1), *wnk1a* morphants (C2), and *wnk1b* morphants (C3) at 33 hpf. (D1) *etv2* expression in uninjected control, *wnk1a* and *wnk1b* morphants as a percentage of embryos exhibit strong or expression, (D2) ISV length in *wnk1a* and *wnk1b* morphants.

To further verify that *wnk1* functions in angiogenesis rather than vasculogenesis, we examined the expression of the vasculogenesis marker *etv2* in somite matched control, *wnk1a and wnk1b* morphants. The embryonic expression of *etv2* is restricted to the earliest precursors of vascular endothelial cells. Morpholino knockdown of *etv2* results in the absence of vasculogenesis [51]. *wnk1a* and *wnk1b* morpholinos were injected into *fli1:GFP* embryos, and the effect on ISVs was assayed. We found that there is no difference in *etv2* expression between the *wnk1a* or *wnk1b* morphants and control embryos at 14 hpf (Fig. 5A1– A3) and 18 hpf (Fig. 5B1– B3), whereas the development of ISVs is significantly affected (Fig. 5C1–C3). The quantified results for *etv2* expression at 18 hpf and the length of ISVs at 33 hpf were shown in Figure 5D1 and D2 respectively.

### Knockdown of endothelial-specific *pi3kc2α* inhibits the growth of intersegmental vessels

Previous studies have demonstrated that VEGF/VEGFR signaling regulates endothelial cell survival and growth through the activation of the PI3K/Akt pathway, and endothelial cell permeability through the activation of endothelial NO synthase (eNOS) [52], [53], [54]. The WNK1 protein contains an Akt phosphorylation motif; therefore, we suspect that Wnk1 participates in angiogenesis after being phosphorylated and activated by Akt kinase downstream of Vegf/Vegfr signaling.

To further analyze whether Wnk1 acts through the Vegf/Vegfr2-PI3K/Akt pathway in endothelial cell activation, we wanted to inhibit PI3K, which falls between Vegfr2 and Wnk1 in the proposed signaling cascade, to check the integrity of this pathway in angiogenesis. To date, there are eight known PI3K isoforms with varying structural features and lipid substrate preferences; they are divided into three classes (class I, class II and class III), and these isoforms are involved in metabolic control, immunity, angiogenesis and cardiovascular homeostasis [55]. From the literature, we know that endothelial cells only express PI3KC2α [56]. From an NCBI GenBank search, we found that the zebrafish gene phosphoinositide-3-kinase, class 2, alpha polypeptide (*pik3c2a)* is similar to the human *PI3KC2α* homologs. The *pi3kc2α* mRNA expression pattern from ZFIN (Bernard Thisse’s group, Expression of the zebrafish genome during embryogenesis. ZFIN Direct Data Submission (http://zfin.org/cgi-bin/webdriver?MIval=aa-ZDB_home.apg)) showed that *pi3kc2α* is expressed in axial vasculature at 24 hpf (Fig. S4), providing support for a role for *pi3kc2α* in angiogenesis.

**Figure 6:**
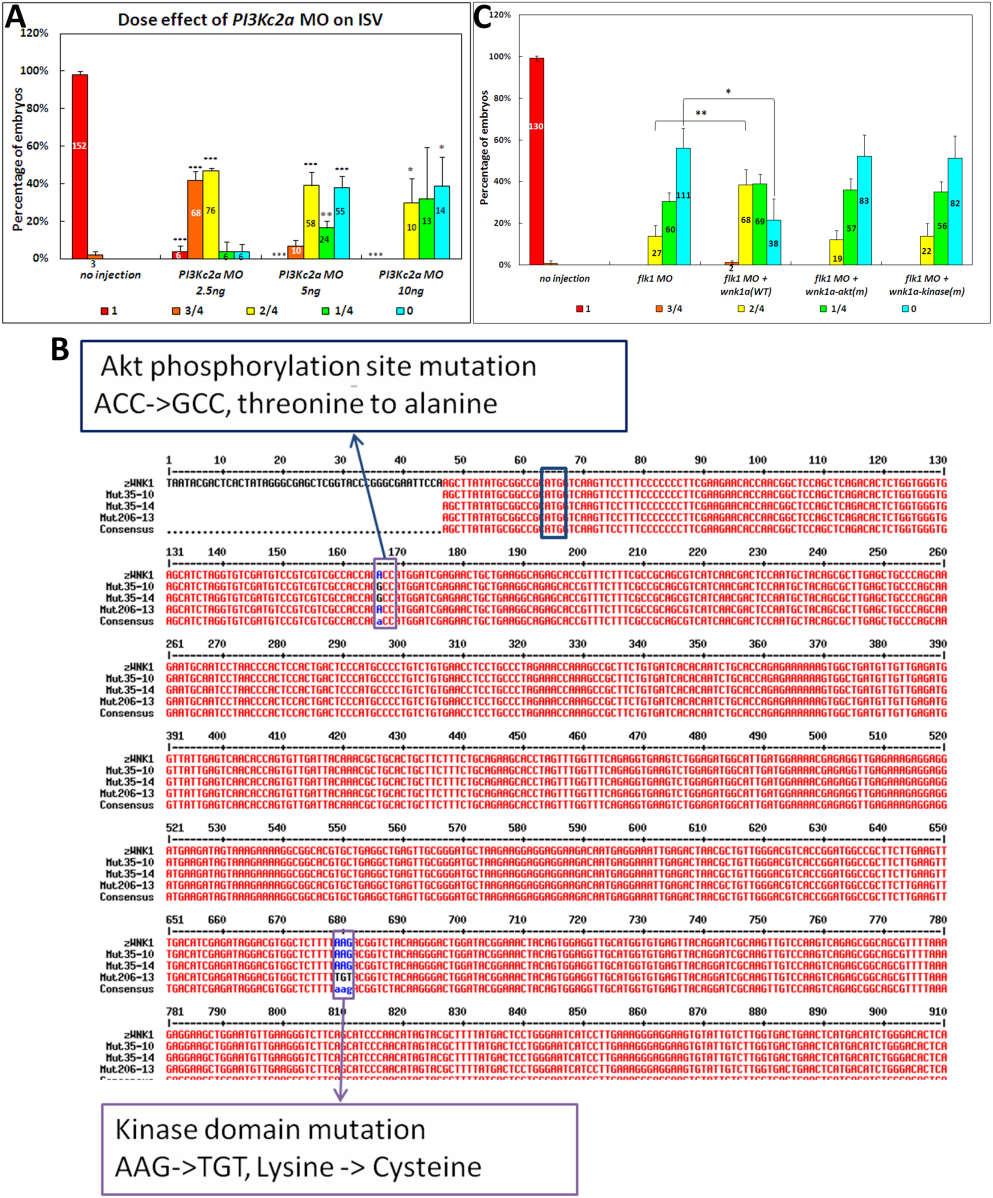
Effect of knockdown of the PI3K ortholog on ISVs and the rescue effects of wild-type *wnk1a*, *wnk1a* containing an Akt phosphorylation site mutation and kinase-deficient *wnk1a* on *flk1* morphants. (A) The effects of *pi3kc2α* MO on the length of intersegmental vessels. (B) Two Thr35 mutations and one Lys206 mutation were generated using site-directed mutagenesis. The sequence of wild-type *wnk1a* aligned with Thr35 and Lys206 mutants, which are Akt phosphorylation site and kinase domain mutants, respectively. (C) Co-injection of *wnk1a* mRNA rescues *flk1* morphants. Quantitative analysis of the length of the ISVs in *flk1* morphants co-injected with various *wnk1a* mRNAs. Experiments were performed at least three times, and the number of embryos analyzed is shown in the bar graph. Red indicates that the ISVs grew to full length, orange indicates that the ISVs were 75% of the normal length, yellow indicates 50%, green indicates 25%, and light blue indicates no ISVs were observed in the embryos. The differences between treatments were assessed using a two-tailed Student’s *t*-test. Significant differences between the morphants and controls are indicated (_*_, *P*<0.05; _**_, *P*<0.01; and _***_, *P*<0.001).

We then designed a MO to knockdown *pik3c2a*. The *pi3kc2α* morphant exhibits growth inhibition in the ISVs similar to that observed in the *vegfr2*, *wnk1a* and *wnk1b* morphants (Fig. 6A), suggesting that Pi3kc2α and Wnk1 may be part of the same pathway. We hypothesized that upon Vegf binding to Vegfr, Pi3kc2α is activated, which phosphorylates and activates Wnk1, which in turn activates downstream targets. Additionally, we found that injection of 10 ng of *pi3kc2α* MO into embryos significantly increases mortality relative to controls, suggesting that Pi3kc2α might participate in other physiological functions, possibly through other signaling pathways. The number of embryos examined and the phenotypic and mortality analyses of *pi3kc2a* morphants versus other morphants are provided in Fig. S5.

### Partial Rescue of the angiogenesis defect in *vegfr2* morphants by wild-type, but not Akt-phosphorylation site mutant or kinase-dead mutant, *wnk1a* mRNA

Based on the results presented in Figure 3 and 6A, we hypothesized that the binding of Vegf to Vegfr2 activates Wnk1a through phosphorylation by the PI3K-Akt kinase cascade. To test this theory, we used site-directed mutagenesis to generate two mutants of Wnk1a, one missing the Akt phosphorylation site and the other lacking kinase activity (Fig. 6B). We transcribed mRNA for wild-type *wnk1a* and the two mutants *in* *vitro* and then co-injected these with the *vegfr2* MO for rescue experiments.

Figure 6C illustrates the result of the rescue experiments. Co-injection of *vegfr2* MO with wild-type *wnk1a* RNA, but not *wnk1a-akt(m)* or *wnk1a-kinase(m),* leads to a statistically significant increase in angiogenesis compared with vegfr2 MO injection alone. Representative images of rescued morphants are provided in Figure S6. Notably, the rescue is incomplete: ISVs in the *vegfr2* morphants rescued with *wnk1a* RNA are only half as long as in wildtype. It is possible that some Vegfr2 activity remains in morphant embryos, and hence wild-type Wnk1 can be activated and subsequently phosphorylate downstream targets to achieve rescue. Therefore, the rescue is partially due to the morpholino knockdown the Vegfr2.

**Figure 7:**
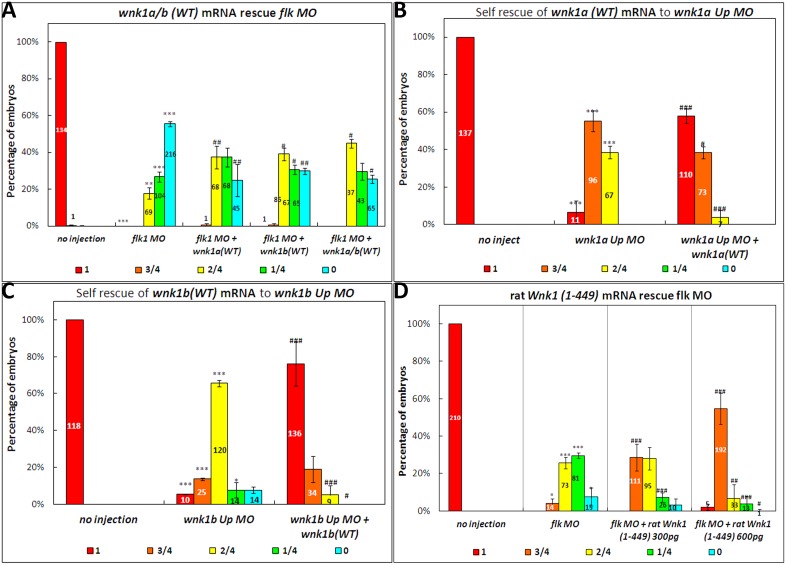
Effect of wild-type *wnk1a* and *wnk1b* mRNA injection on *flk1*, *wnk1a* and *wnk1b* morphants. (A) Co-injection of *wnk1a*, *wnk1b* or both *wnk1a* and *wnk1b* mRNA rescues *flk1* morphants. (B) Co-injection of *wnk1a* mRNA rescues the ISV defect caused by the *wnk1a* upstream MO. (C) Co-injection of *wnk1b* mRNA rescues the ISV defect caused by the *wnk1b* upstream MO. (D) Injection of rat *Wnk1(1–449)* rescues *flk1* morphants. Experiments were performed at least three times, and the number of embryos analyzed is shown in the bar graph. Red indicates that the ISVs grew to full length, orange indicates that the ISVs were 75% of the normal length, yellow indicates 50%, green indicates 25%, and light blue indicates no ISVs were observed in the embryos. The differences between treatments were assessed using a two-tailed Student’s *t*-test. Significant differences between the morphants and controls are indicated (_*_, *P*<0.05; _**_, *P*<0.01; and _***_, *P*<0.001); significant differences between co-injection of RNA with morpholino and morpholino alone are also indicated (_#_, *P*<0.05; _##_, *P*<0.01; and _###_, *P*<0.001).

Because there are two Wnk1 isoforms in zebrafish, we wondered if the ISVs could be completely rescued by providing both isoforms. Co-injection of both *wnk1a* and *wnk1b* mRNA with *flk1* MO, however, is comparable to co-injection with *wnk1a* or *wnk1b* RNA alone (Fig. 7A). Wild-type *wnk1a* and *wnk1b* mRNA fully rescue the angiogenesis defects in *wnk1a* and *wnk1b* morphants, respectively (Fig. 7B and 7C). Thus, the partial rescue is not due to poor quality or low abundance of the mRNA.

Because no biochemical assays have been performed to confirm the expected effects of truncation on zebrafish *wnk1a* and *wnk1b*, we used a constitutively active rat Wnk1 truncation, for which biochemical data are available, in rescue assays. Previous biochemical studies on rat Wnk1 have demonstrated that the Wnk1(1–449) truncation is constitutively active due to the lack of an autoinhibitory domain [10]. The truncated form of Wnk1 was expected to be better able to rescue *flk1* morphants than wild-type Wnk1. We obtained rat Wnk1 cDNA from Dr. Cobb’s lab, subcloned the Wnk1(1–449) fragment and then generated mRNA for co-injection with *flk1* MOs. We demonstrated that rat Wnk1(1–449) effectively rescues ISV formation in *flk1* morphants in a dose-dependent manner (Fig. 7D).

### Knockdown of *flk1 or flt4* decreases expression of *wnk1* mRNA

**Figure 8:**
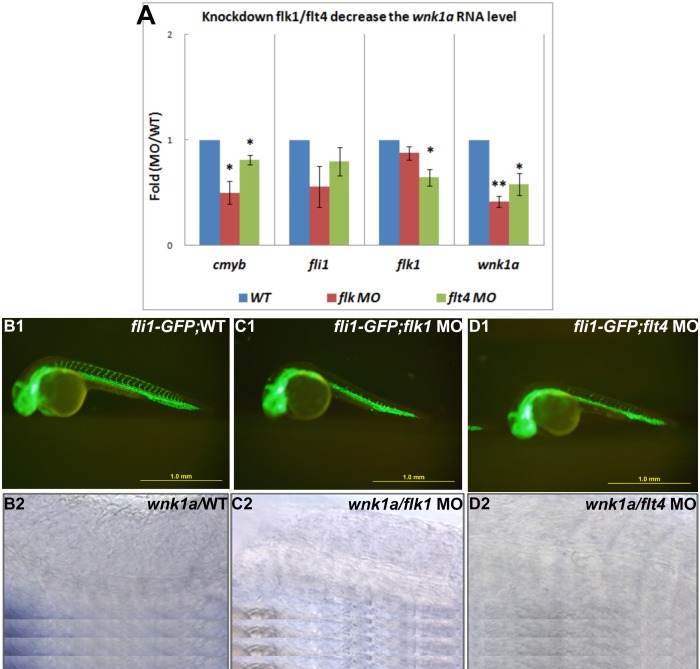
Injections of *flk1* MO and *flt4* MO decrease *wnk1* mRNA expression. (A) Relative fold-change comparisons between *flk1* and *flt4* morphants and uninjected controls. Comparison of mRNA expression levels at 33 hpf for important transcription factors, receptors, and *wnk1*. Blue, red, and green bars denote mRNA expression in wild-type embryos, *flk1* morphants and *flt4* morphants, respectively. The x-axis indicates the expressed genes, and the y-axis shows the fold differences between the morphants and the control. The differences between treatments were assessed using a two-tailed Student’s *t*-test. Significant differences between the morphants and the controls are indicated (_*_, *P*<0.05 and_**_, *P*<0.01). (B1, C1, D1) The ISVs are affected in morphants compared with wild-type embryos. (B2, C2 and D2) The expression of *wnk1a* is reduced in the PCV in *flk1* and *flt4* morphants. *wnk1a* mRNA was detected with *in* *situ* hybridization in wild-type embryos (B2), *flk1* morphants (C2) and *flt4* morphants (D2).

Another possible mechanism to explain how VEGF signal transduction affects angiogenesis through *wnk1* involves transcriptional regulation. VEGF signal transduction might also regulate *wnk1* gene expression. The co-localization of *flt4* (vegfr3) and *wnk1* in the PCV strengthens this possibility. Furthermore, we have analyzed *flk1* and *flt4* morphants for *wnk1* expression. q-RT-PCR analysis of *wnk1a* and other endothelial genes demonstrated a significant reduction in *wnk1a* RNA in *flk1* and *flt4* morphants relative to controls (Fig. 8A). Several genes for blood/vessel formation (*cmyb*, *fli1* and *flk1*) are also down regulated in *flk1* and *flt4* morphants. ISVs are inhibited by *flk1* and *flt4* MO injection (Fig. 8B1, C1 and D1) and expression of *wnk1a* in the PCV is decreased in *flk1* and *flt4* morphants compared with uninjected controls (Fig. 8 B2, C2 and D2).

## Discussion

In this study, we found that knockdown of *vegfr2*, *vegfr3*, *wnk1* or *pi3kc2a* causes angiogenesis defects. From published works, we also know that Wnk1 can be phosphorylated by Akt kinase, and our zebrafish Wnk1a protein sequence contains an Akt phosphorylation site. Therefore, we propose that Wnk1a is downstream of the Vegfr2/Fkl1-PI3K-Akt signaling pathway in zebrafish. In *wnk1a* mRNA rescue experiments, we found that wild-type *wnk1a*, but not *wnk1a* with an Akt phosphorylation site mutation, partially rescues the angiogenesis defect of *vegfr2* knockdown. The finding that the Akt site mutant fails to rescue the angiogenesis defect of *flk1* morphants supports the hypothesis that binding of Vegf to Vegfr2 activates PI3K and Akt kinase, which then phosphorylate Wnk1 to regulate angiogenesis. In addition, we found that rat Wnk1(1–449), a constitutively active form that lacks the autoinhibitory domain, effectively rescues the *flk1* morphant. One important question arising from these results, though, is why wild-type *wnk1a* RNA rescues *flk1* morphants if the function of Vegfr2/Flk1 pathway is to phosphorylate and activate Wnk1.

To address this issue, we postulated that the Vegfr2 pathway might also regulate the expression of *wnk1a*. We performed real-time q-RT-PCR and *in* *situ* hybridization to investigate this possibility. Our q-RT-PCR results reveal that knockdown of either *flk1* or *flt4* causes a decrease in the *wnk1a* mRNA level. The reduction in *wnk1a* expression is modest, perhaps because *wnk1a* is broadly expressed in a variety of tissues, including some not affected by the relatively vascular-specific knockdown of *flk1* or *flt4*. The *in* *situ* hybridization results also suggest that the expression of *wnk1a* in the PCV is decreased in *flk1* and *flt4* morphants. Together, the q-RT-PCR and *in* *situ* results support the notion that the expression of *wnk*1a is down regulated by knockdown of *flk1* and *flt4*. This downregulation of *wnk1a* would explain why co-injection of wild-type *wnk1a* RNA rescues the angiogenesis defect of *fkl1* morphants. Because morpholino knockdown is incomplete, residual Flk1 protein must remain in *flk1* morphants. It is conceivable that overexpression of Wnk1a proteins (by mRNA injection) can partially overcome decreased phosphorylation of Wnk1a by the Flk1-PI3K-Akt cascade. How Vegfr2/Flk1 regulates *wnk1a* expression remains unknown and awaits future investigation.

Angiogenesis comprises four main steps: selection of sprouting endothelial cells, sprout outgrowth and guidance, sprout fusion, and perfusion/maturation [49]. These processes are regulated by different growth factors, receptors and downstream signal molecules. In the *wnk1a* morphants, we observed that the number of endothelial cells participating in the ISVs and the intervals between ISVs are similar to those in wild-type embryos. Knockdown of *wnk1* suppresses the growth of the ISV toward the DLAV. We also found that ISVs become straighter and finer in embryos overexpressing *wnk1a* mRNA than in wild-type embryos (Fig. S5). Therefore, we speculate that Wnk1a may be involved in sprout outgrowth, migration and elongation of the selected tip cells, rather than in the selection of sprouting endothelial cells or lateral inhibition among endothelial cells. We speculate that Wnk1a has no interaction with the Notch signaling pathway, which is involved in lateral inhibition.

The expression pattern of *WNK1* is ubiquitous in human, mouse and zebrafish. In a previous study [22], it was found that *Wnk1* mRNA is expressed in endothelial tissue such as the heart, the brachial arches, the dorsal aorta, pericytes and other manifestations of the cardiovascular system. *Wnk1* is also expressed in the neural tube and the gut. From our *in* *situ* data, we found the zebrafish *wnk1* is expressed in the neural tube, notochord, and the vascular structure-posterior cardinal vein (PCV). In *Wnk1*-null mice, *Wnk1* mRNA was absent in all these tissues. Using a Cre recombinase system, endothelial-specific Wnk1 expression rescues angiogenesis in *Wnk1*-null mice. This result suggests that *Wnk1* expression in tissues beyond the vascular endothelial cells is not necessary for angiogenesis. Although we have not shown that endothelial-specific *wnk1* can rescue angiogenesis in zebrafish *wnk1* null mutants, we believe the function of *wnk1* in angiogenesis is highly conserved among vertebrates. Only endothelial-specific expression of *wnk1* can rescue angiogenesis in the *Wnk1* ^–/–^ background [22].

VEGF is an important factor in promoting angiogenesis. Compared with normal tissues, most tumors grow faster *in* *vivo* and often require additional blood to supply the necessary nutrients and oxygen; thus, many tumors secrete various angiogenic factors and induce vascular endothelial cells to promote proliferation and tumor expansion. In the healthy body, the role of VEGF is limited mainly to wound healing and menstruation. However, in the process of tumor growth and metastasis, VEGF and downstream genes play an indispensable role. Therefore, VEGF therapies that inhibit the formation of new blood vessels have become very important in cancer treatment. In future work, we will xenotransplant cancer cells into *Tg(fli1:EGFP)* zebrafish to observe angiogenesis occurring near the cancer cells in live animals. Using drugs that inhibit VEGF as well as the *wnk1* morpholino antisense oligonucleotides, we will test the feasibility of using anti-*wnk1* MOs to inhibit angiogenesis, tumor growth and metastasis.

## Supporting Information

Figure S1 **Morpholino specificity revealed by co-injections of** ***wnk1a-GFP*** **or** ***wnk1b-GFP*** **with various morpholinos.** (A1, B1) Schematic of *wnk1a-GFP* and *wnk1b-GFP* constructs and the location of morpholino target sites. (A2, B2) Injection of *wnk1a-GFP* or *wnk1b-GFP* only. Co-injection of *wnk1a-GFP* or *wnk1b-GFP* with (A3, B3) *wnk1a* or *wnk1b* MOs targeted to the ATG, (A4, B4) *wnk1a* or *wnk1b* MOs that bind upstream of the translation start site, (A5, B5) scrambled control MOs, (B6) MOs targeted to the other isoform’s ATG, and (A6, A7) MOs targeted to the 5′ untranslated region of the other isoform.
(TIF)

Figure S2 **Temporal expression patterns of** ***wnk1a and wnk1b.*** Whole mount *in* *situ* hybridization to detect *wnk1a* (A1∼A12) and *wnk1b* (B1∼B12) mRNA expression was performed at the indicated time points. Whole mount *in* *situ* hybridization of sense probes for *wnk1a* (A13) and *wnk1b* (B13) at 48 hpf showed no signal. All pictures are lateral views. Scale bar: 100 µm.
(TIF)

Figure S3 **Measurement of the length of ISVs and representative images of phenotypic classification.** (A1) Illustration of the region used to measure the ISVs. (A2∼A6) ISVs categorized as having extended over 100%, 75%, 50%, 25% or 0% of the distance from the DA (or PCV) to the DLAV at 33 hpf. (B1∼B4) Phenotypes were characterized as normal, class 1, class 2 or class 3 at 24 hpf.
(TIF)

Figure S4 **Whole mount** ***in*** ***situ*** **hybridization for** ***pi3kc2a*** **mRNA at the indicated time points. Images were obtained from ZFIN.**
(TIF)

Figure S5 **Phenotypic classification of 24 hpf** ***flk1*** **morphants.** *flt4* morphant (A), *pi3kc2a* morphant (B), *wnk1a* morphant (C), *wnk1a* UP morphant (D) and *wnk1a* 5 MM morphant (F). For each morphant, there are two figures. The first figure shows the number of embryos analyzed, and the second figure shows the percentage of morphants displaying a phenotype.
(TIF)

Figure S6 **Phenotype of** ***Tg(fli1:GFP)*** **embryos injected with various morpholinos and imaged with a florescence microscope.** (A–E) Uninjected control embryos (A), *flk1* morphants (B) and *flk1* morphants co-injected with wnk1a (WT) mRNA (C), *wnk1a*-akt(m) mRNA (D), or *wnk1a*-kinase(m) mRNA (E) at 33 hpf. (F–H) Frontal views of the heads of uninjected control embryos (F), *wnk1b* morphants (G) or *wnk1b* morphants co-injected with *wnk1b* mRNA at 33 hpf. (I and J) *wnk1a* mRNA injected embryo (I) compared to wild-type control (J) at 29 hpf.
(TIF)

Data S1 **Detailed information regarding q-RT-PCR primers,** ***in*** ***situ*** **probe location, morpholino design, and** ***wnk1*** **-GFP constructs.**
(DOCX)
