# Supplementary figures and images for: Zebrafish WNK Lysine Deficient Protein Kinase 1 (wnk1) Affects Angiogenesis Associated with VEGF Signaling
Source: PLoS One. 2014 Aug 29;9(8):e106129. doi: 10.1371/journal.pone.0106129 (PMC4149531; doi:10.1371/journal.pone.0106129)

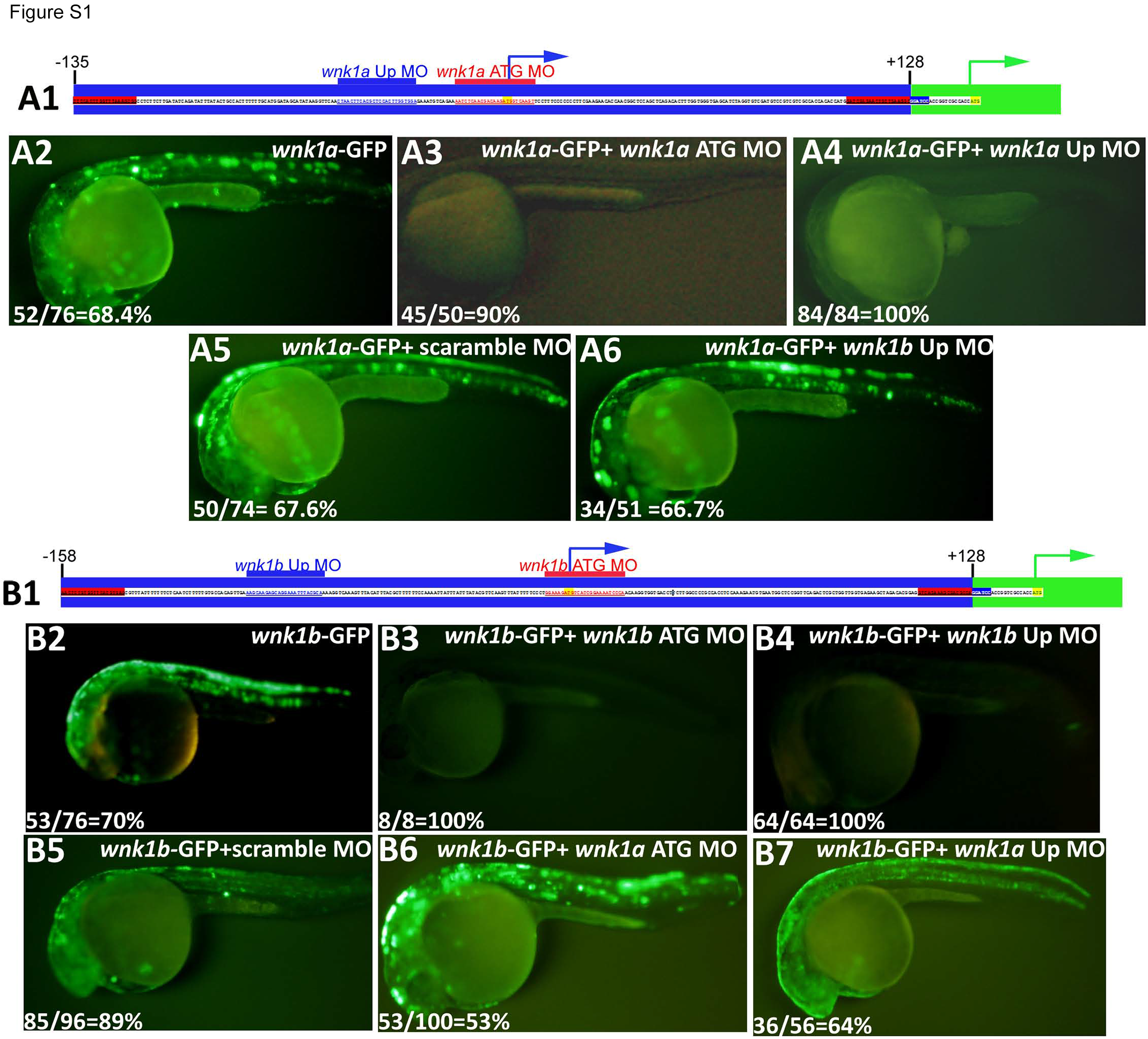

Supplement: Figure S1 — Morpholino specificity revealed by co-injections of wnk1a-GFP or wnk1b-GFP with various morpholinos. (A1, B1) Schematic of wnk1a-GFP and wnk1b-GFP constructs and the location of morpholino target sites. (A2, B2) Injection of wnk1a-GFP or wnk1b-GFP only. Co-injection of wnk1a-GFP or wnk1b-GFP with (A3, B3) wnk1a or wnk1b MOs targeted to the ATG, (A4, B4) wnk1a or wnk1b MOs that bind upstream of the translation start site, (A5, B5) scrambled control MOs, (B6) MOs targeted to the other isoform’s ATG, and (A6, A7) MOs targeted to the 5′ untranslated region of the other isoform. (TIF) [file pone.0106129.s001.tif]

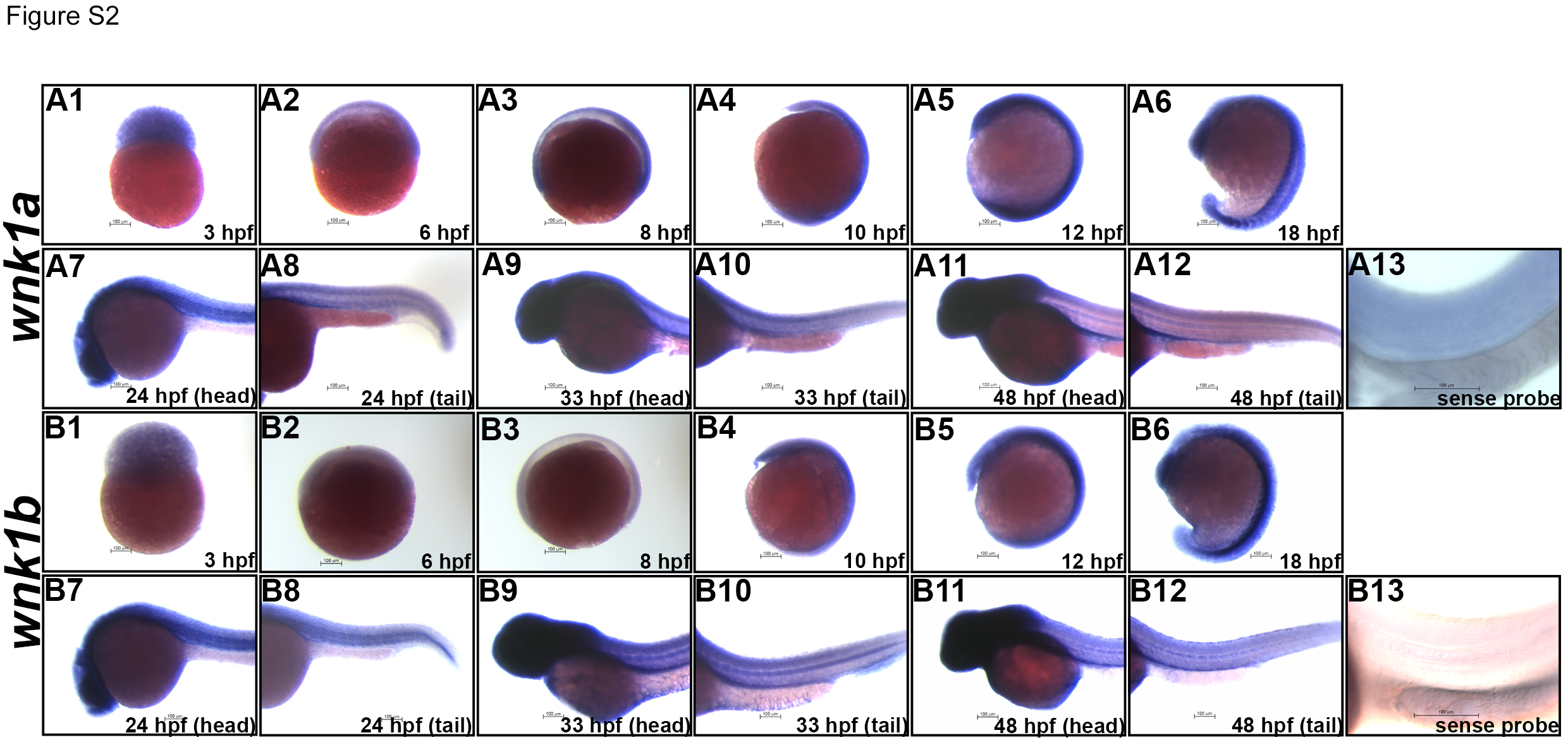

Supplement: Figure S2 — Temporal expression patterns of wnk1a and wnk1b. Whole mount in situ hybridization to detect wnk1a (A1∼A12) and wnk1b (B1∼B12) mRNA expression was performed at the indicated time points. Whole mount in situ hybridization of sense probes for wnk1a (A13) and wnk1b (B13) at 48 hpf showed no signal. All pictures are lateral views. Scale bar: 100 µm. (TIF) [file pone.0106129.s002.tif]

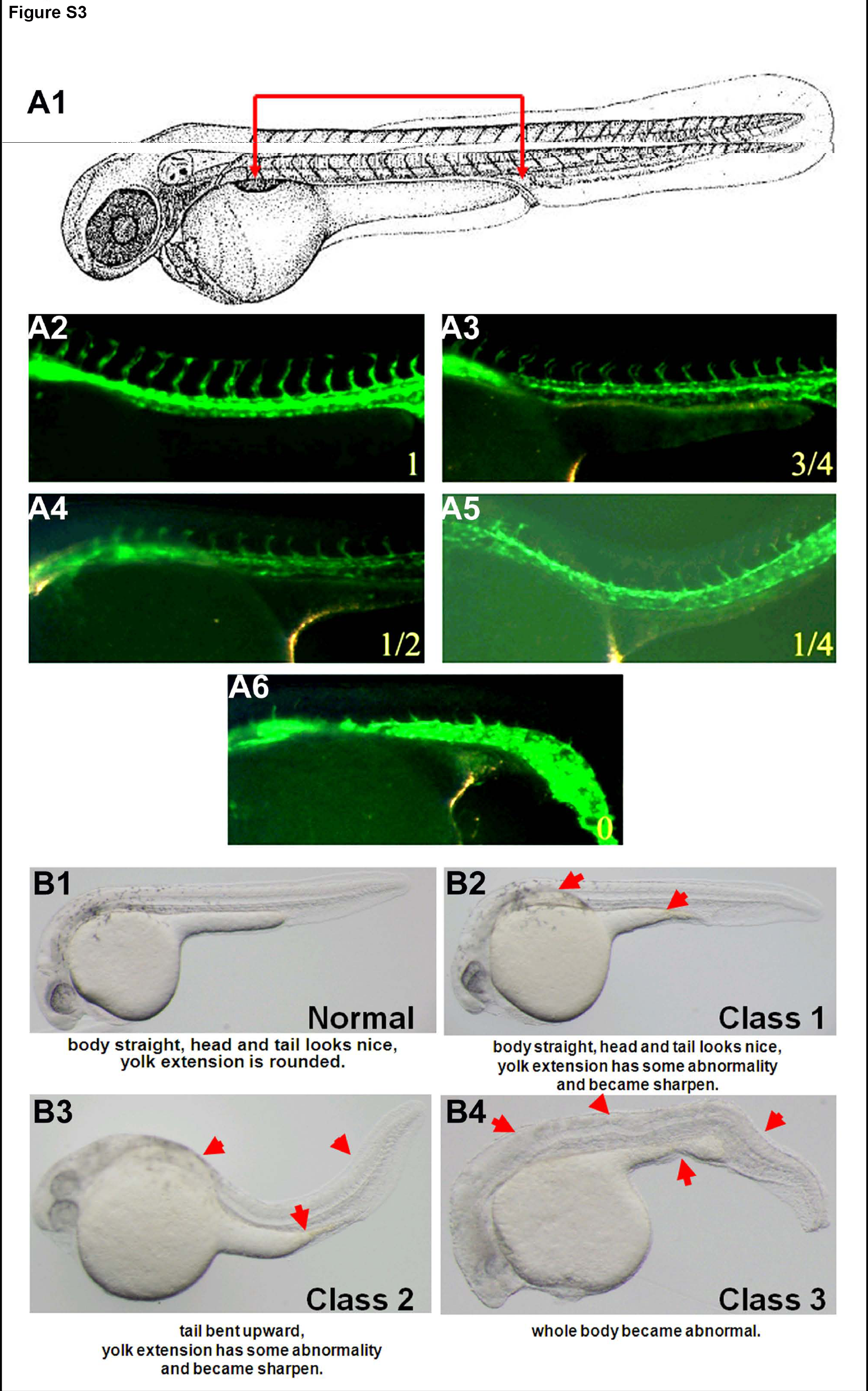

Supplement: Figure S3 — Measurement of the length of ISVs and representative images of phenotypic classification. (A1) Illustration of the region used to measure the ISVs. (A2∼A6) ISVs categorized as having extended over 100%, 75%, 50%, 25% or 0% of the distance from the DA (or PCV) to the DLAV at 33 hpf. (B1∼B4) Phenotypes were characterized as normal, class 1, class 2 or class 3 at 24 hpf. (TIF) [file pone.0106129.s003.tif]

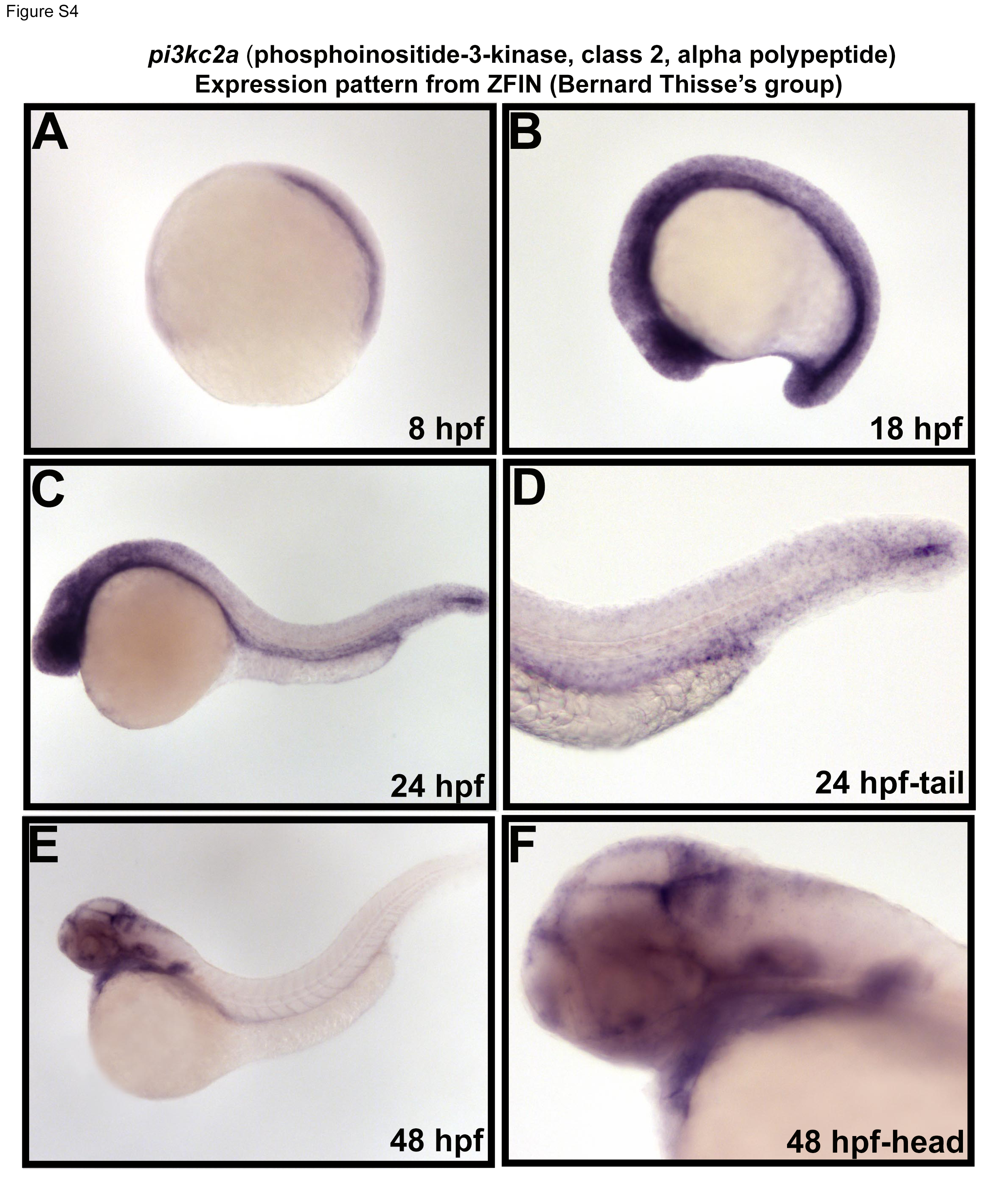

Supplement: Figure S4 — Whole mount in situ hybridization for pi3kc2a mRNA at the indicated time points. Images were obtained from ZFIN. (TIF) [file pone.0106129.s004.tif]

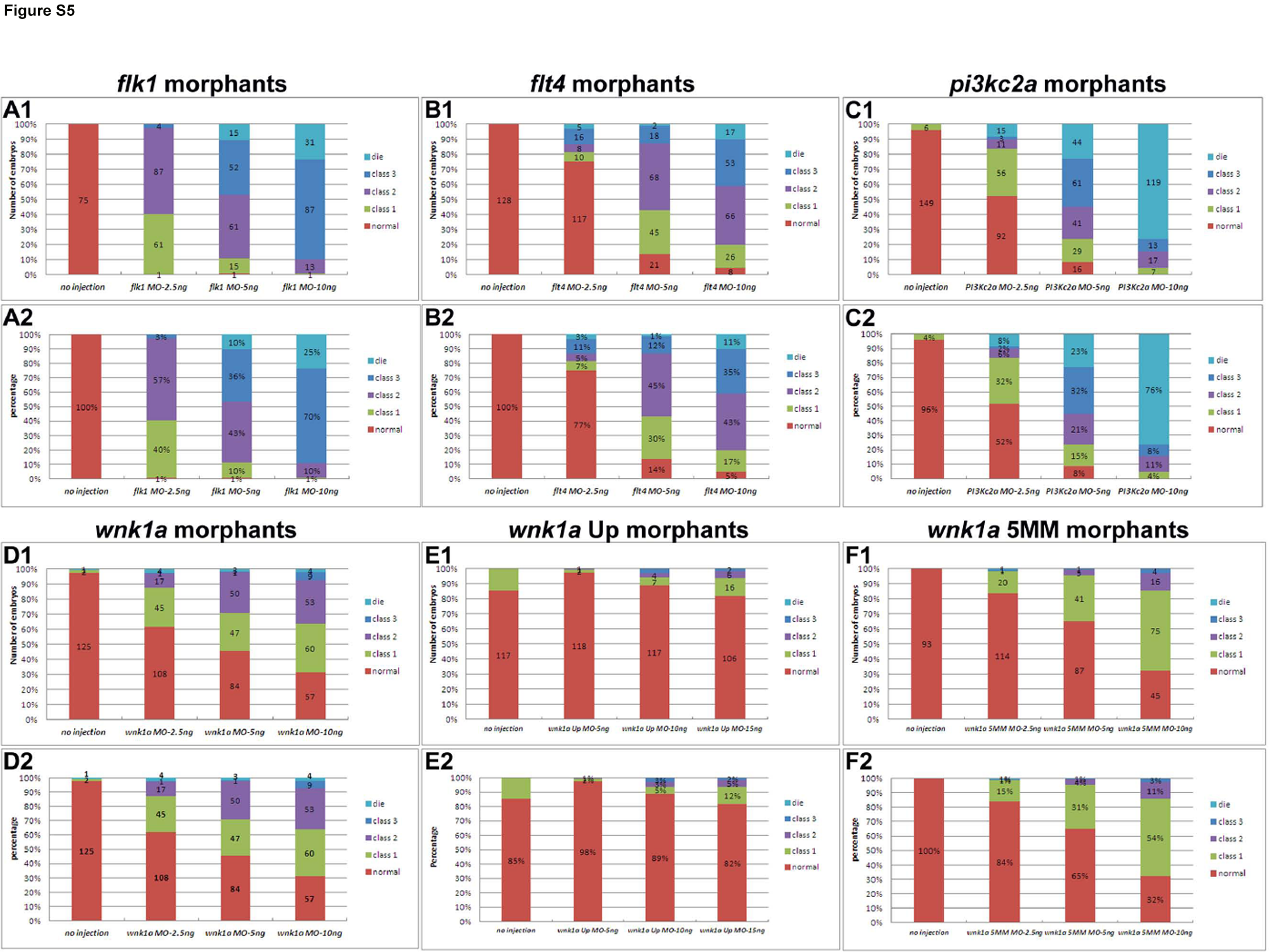

Supplement: Figure S5 — Phenotypic classification of 24 hpf flk1 morphants. flt4 morphant (A), pi3kc2a morphant (B), wnk1a morphant (C), wnk1a UP morphant (D) and wnk1a 5 MM morphant (F). For each morphant, there are two figures. The first figure shows the number of embryos analyzed, and the second figure shows the percentage of morphants displaying a phenotype. (TIF) [file pone.0106129.s005.tif]

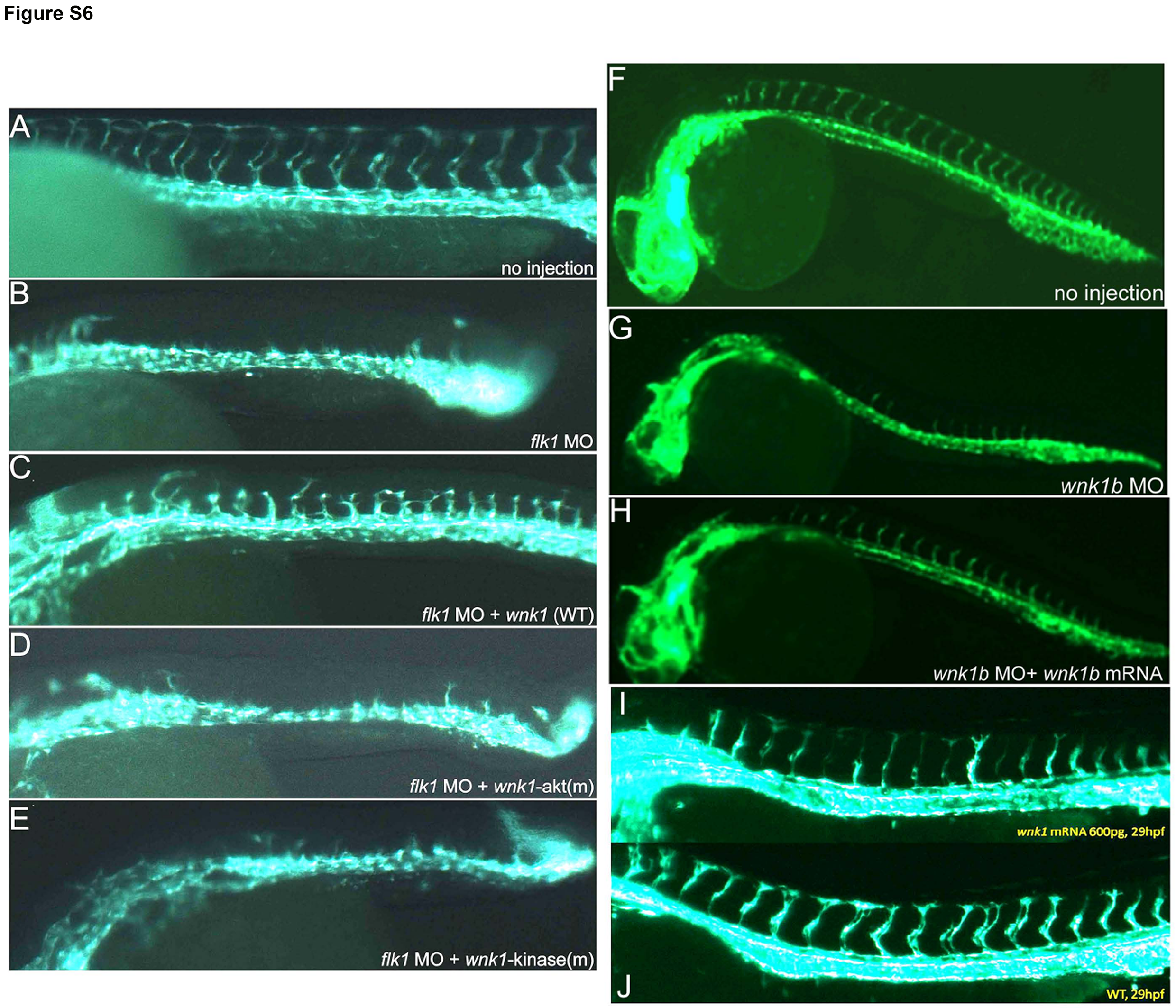

Supplement: Figure S6 — Phenotype of Tg(fli1:GFP) embryos injected with various morpholinos and imaged with a florescence microscope. (A–E) Uninjected control embryos (A), flk1 morphants (B) and flk1 morphants co-injected with wnk1a (WT) mRNA (C), wnk1a-akt(m) mRNA (D), or wnk1a-kinase(m) mRNA (E) at 33 hpf. (F–H) Frontal views of the heads of uninjected control embryos (F), wnk1b morphants (G) or wnk1b morphants co-injected with wnk1b mRNA at 33 hpf. (I and J) wnk1a mRNA injected embryo (I) compared to wild-type control (J) at 29 hpf. (TIF) [file pone.0106129.s006.tif]
