# Supplementary material for: Zebrafish WNK Lysine Deficient Protein Kinase 1 (wnk1) Affects Angiogenesis Associated with VEGF Signaling
Source: PLoS One. 2014 Aug 29;9(8):e106129. doi: 10.1371/journal.pone.0106129 (PMC4149531; doi:10.1371/journal.pone.0106129)
Supplement: Data S1 — Detailed information regarding q-RT-PCR primers, in situ probe location, morpholino design, and wnk1 -GFP constructs. (DOCX) [file pone.0106129.s007.docx]

**Supporting Data.**

**Q-RT-PCR primer and in-situ probes’ location and Morpholinos design**

***wnk1a* in situ primer design (965 bp)**

*wnk1a*-T3-Forward: **^5’^**AATTAACCCTCACTAAAGGGAGA**CACAAGCCTCAAATCAGCAA^3’^**

*wnk1a*-T7-Reverse: **^5’^**TAATACGACTCACTATAGGGAGA**AGATGGTGTTTTGCCCAGAC^3’^**

(reverse complementary: **^5’^GTCTGGGCAAAACACCATCT^3’^**)

***wnk1a* QPCR primer (201 bp)**

*wnk1a*-QPCR-forward: **^5’^**TCGAGATAGGACGTGGCTCT**^3’^**

*wnk1a*-QPCR-reverse: **^5’^**TCAAGGATGATTCCCAGGAG**^3’^**

(reverse complementary: **^5’^**CTCCTGGGAATCATCCTTGA**^3’^**)

***wnk1a* ATG-MO:** **^5’^ACTTGACCATCTTGTCGTTGAGATT^3’^**

(reversecomplementary: **^5’^AATCTCAACGACAAGATGGTCAAGT^3’^)**

*wnk1*-5 MM MO: **^5’^**ACTTCACGATCTTCTCCTTGACATT**^3’^**

***wnk1a* Up MO:** **^5’^TCCACCAAGTGGAGCGTGAAGTTAG^3’^**

(reversecomplementary: **^5’^CTAACTTCACGCTCCACTTGGTGGA^3’^)**

>*wnk1a* Contiq sequence:

TTCCACTTGGTTTAAAGCGGCCTCTTCTTGATATCAGATATTTATACTGCCACTTTTTTGCATGGATAGCATATAAGGTTCAA**CTAACTTCACGCTCCACTTGGTGGA**GAAATGTCAGAA**AATCTCAACGACAAGATGGTCAAGT**TCCTTTCCCCCCCTTCGAAGAACACCAACGGCTCCAGCTCAGACACTCTGGTGGGTGAGCATCTAGGTGTCGATGTCCGTCGTCGCCACCACACCATGGATCGAGAACTGCTGAAGGCAGAGCACCGTTTCTTTCGCCGCAGCGTCATCAACGACTCCAATGCTACAGCGCTTGAGCTGCCCAGCAAGAATGCAATCCTAACCCACTCCACTGACTCCCATGCCCCTGTCTGTGAACCTCCTGCCCTAGAAACCAAAGCCGCTTCTGTGATCACACAATCTGCACCAGAGAAAAAAGTGGCTGATGTTGTTGAGATGGTTATTGAGTCAACACCAGTGTTGATTACAAACGCTGCACTGCTTCTTTCTGCAGAAGCACCTAGTTTGGTTTCAGAGGTGAAGTCTGGAGATGGCATTGATGGAAAACGAGAGGTTGAGAAAGAGGAGGATGAAGATAGTAAAGAAAAGGCGGCACGTGCTGAGGCTGAGTTGCGGGATGCTAAGAAGGAGGAGGAAGACAATGAGGAAATTGAGACTAACGCTGTTGGGACGTCACCGGATGGCCGCTTCTTGAAGTTTGACATCGAGATAGGACGTGGCTCTTTTAAGACGGTCTACAAGGGACTGGATACGGAAACTACAGTGGAGGTTGCATGGTGTGAGTTACAGGATCGCAAGTTGTCCAAGTCAGAGCGGCAGCGTTTTAAAGAGGAAGCTGGAATGTTGAAGGGTCTTCAGCATCCCAACATAGTACGCTTTTATGACTCCTGGGAATCATCCTTGAAAGGGAGGAAGTGTATTGTCTTGGTGACTGAACTCATGACATCTGGGACACTCAAGACGTATCTAAAACGGTTTAAGGAGATGAAGATCAAAGTCTTGCGCAGCTGGTGCCGGCAGATTTTGAAGGGCCTACACTTCCTTCATACCAGGTCACCACCTATCATCCACCGGGATCTGAAGTGTGACAACATCTTCATCACTGGGCCTACTGGATCAGTCAAGATTGGAGATTTGGGCCTTGCGACCCTCAAGAGGGCCTCCTTTGCTAAGAGCGTCATAGGTACCCCTGAGTTCATGGCGCCTGAGATGTATGAAGAGAAGTATGACGAGTCGGTGGATGTGTATGCCTTTGGGATGTGTATGCTGGAGATGGCTACTTCTGAGTACCCGTACTCCGAGTGTCAGAATGCAGCTCAGATCTATCGCAGAGTCACCAGCGGGGTGAAGCCTGGCAGTTTTGACAAAGTGGCTATTCCAGAGGTGAAGGAGATCATTGAGGGTTGCATTcGTCAGAATAAAGATGAGAGGTAcTGCATCAAGGATcTCcTAAGCCATgcCtTcTTCCAggAGGAGAcTGGAGTTCGTGTAGAACTGGCTGAGGAGGATGATGGAGAGCTTGTGGCTATAAAGTTGTtGcTGCGCATTGAgGATGTTAAGAAACTTAAAGGCAAATACAAAGAGAATGAAGCCATCGAGTTTTCcTTcGACTTGGATAAAGACGTCCCAGATGATGTGGCTCAAGAAATGGTTGAATCAGGCTACATTTGTGAGGGTGACCaCAAAACCATTGCAAAGGCCATAAAGGACAGAGTGTCCcTGATCTCCCGTAAGAGAGAGCAGAGAAAACTGGTGAGAGAAGAACAGGAAAAAAGGAAGATGGAGCAGGAGAATGAcGCCGCCCAGCAAACTAACCCCACAGTCAAGTCCCCTGGTTCTGcTcTGGCcTCAGTCATAATGGAGTCTGAAGAAGCAGAAACAGACCAGCACCAGCAGCCTGGAGCCTCTGCTTCTGCTGTAGGGTTTGTGGATCCTCAAAGTTCAGTGACcATCCTcGAGGCTCATCCTGTCCAGCCAAACACATCTTTCAGCACCACACAGCCTGATCCGCAGCCACAGCACACAATTATACGTCCACAAAGCATGTCGCAGCACCCAGTCCAGGCCCCTCTGCAGCAGCCGAGCAGTACTTTGACCCTGGGCCCCGTCCCCCAGACTTCTCCACCTCAGGTGATCGGTCAAACAGCCTCTCTGCCCTCTACTGTTCAATCTATTGTCCCGCTGCAGTACCGTCAGCCACCTCAGGATGGAATCACACTACCTCAATCCCTGCCCAGCGCTCAAGTCTTACAGGCACCACTAAGTGTGTCCCAATCACAACCAGTAGAGCAGACACAACCTCAAACATCTCTGGTTCCTGCCTCCACAGAAAGTGGACACTCTGATCCGGCATCTGGTCTAAGCGATGGGAATGAGGGGAGACACGAGGGTCGCTCTATGAAACGACCCCAACGCCGTTCTGTACGCAGCCGCTCGCGTCATGAAAAAACAGCTAAGGTCAAGCTCAATGTGCTAAATATTTCTAATATTGGAGACAGAGTCGCTGAATGTCAGCTGGAAACCCACAACAGAAAGATGGTCACATTCAAGTTTGATCTGGATGGGGATAATCCTGTAGAAATCGCTGAAATTATGGTCAAAAGTGATTTTATTTTGGAGAGTGAACGAGAGTCCTTCATAGAACAGGTTCAGCTGGTCATTCAGAATGCAGATCAGAAAGGAAAAGCTCCCCAAAATAGCCAAACTCAGGTGATCAGTAATTCGAGCCAACAaGTACCTGAAATTTTAACATCCAGTGTGCCTGGTATGCCACCTAGCTTAGCAGCACaGGTTGTACATTCAGCAGGCAGGCGCTTCATAGTCAGCCCCGTGCCTGAAGCACGACTTCGGGAGCCGTTGTTTGGTACTCCCTCAGCAAACACATCATTTGAAGATACGGCACCAGCACCTGATCCAACATCAGCCAGCAGTGCTCCGCAGGACCAAGCCAACTTTGCCTCTGATTCAAACCAAATTTCTGTGGTACCACCGACACCTGGAGATCTTGCCCCAGAGAGCTCCATTCCACCTACTTCTCAATCACCATACCAGAATCCTGTCCCATCATCCACTGCAACTTTGACTGTGACCACCACTCAAGTAAGCACTCCAACACCAACTACCACTCCGCAACCTATAACAGGGAGGACGTCTCCTTCACCAGCCCCAACAGAACCACCAGCAGCACGTCGTATGTCACTTCCCAGCCCATCCCAGACACCTCAAATTGcTCCAGGAGAGAGTAATGGCTTGGATCAAG**CaCaAGCcTCAAATCAGCAA**GTAACTCAACCGAGTGCAACCGGAGATAGTGAGAGCGACGTACAGGGGAAACCACCTGGAATTGAAGACATTCATGCTcTAGACCAAAAGCTGCGCTCCCTTTTCAAGGACTCATCCCAGAACTCTTGCAGCCAACCTGACGGGTCTGGAGAGACTTCCACAACCTCTCCACCCAACACCATAAGCACTAACACTCCTGGCTTTGTCTCTGGTGTGACGCCTTCTAGTTTGTCGCTCAATTCCAGCGGGAACTTTGCACCTGGTGCTTTCGCCTCCATTGGTACTCCTACAAGCTACACCCAGACACCATCTACCGACCAGTCCCCTCCAAGGCCACAGATTGCTGTTGGTGTAACTCAACCAGATGGACACAACAGTACTGAGGAGAAAGAGCCACAACTTGAGAAAGCTTTCCAACTTGGCCGATTTCAGGTGTCTGTGGCCAGTGATCAAGACCCACCAACTGCTCTAGACCCAGGAGTGGTGGTTTCATCTTCCTCTGCTACCCCATCTTCAACATCATCATCATCATCTTCTTCATCACCATCATCTCTCTCTAGCCCCGAAAACACAATTCATAAATCTCAGACTCCTCCACCCAGAGTCAGCCCTGAACCCGTACCACCTACCACTACAGTTGGCCGCTTTCAGGTGACTTGCAGCACTGACATCAAACTTGGTCGCTTCTCAGTGACACCAGCAGAAAGAAAGTCTTCCTCAGTTGAGGGACCAGACACCGCCAAAGAGCCTAAATTGACCCCTTCCAGTGTGTCCATTACCAATCATTATCTCAGTAGCGACAACGACTCAGAACCAGAAGATGAGGGCTTAAGACGAGAGATGAACAGGCTCAAGGAGAGACATAAGGTTGAGATCATGGCATTGCAGAATAAACAGAAAAAGGAAATTGAGGAGCTCTTCACTC**GTCTGGGCAAAACACCATCT**GTGGTTGTGCCACCCACTGTGGCCATGACGCCTGGTAGACGGAGACCTGCTAAGGGCAAGGGGAACAGGCCAAGAAGTGGTAGTGCCACTCAGGCTGCATCTTTTCAACAAGGGAACAAACAAGCTGTGCAGAGTGGTTCACTCAGTCAGTCAGCCAGTGAGAAGACTTCACCTTCAGAAACAGCACCAAGCTCAAACAATGATGTGCAAGATAATACAAGTCCAGTGAGAAAAGAAAACGGACAGAGCCATGACCAGGACCAAACACCAACAACTAATCTGCCACGCAAGGGCACATTCACAGATGACCTCCATCAGCTGGTTGATAACTGGGCCAGAGATGCCATGAACATGATGCCACAGGTCAAGAAAGGATCCAAAAAGAATGTTCATGAAATGCCACGTAAATACTCCGCACCAGGTCAGCTTTGTTCCACTGGTGGCCACATCCCAGCGACCATCAACCCCACAGCTGGACGGAAGGGTTCAATAGGTGTGGCCAATCAGCAATTTGGCTACACTTGTCCCAACTACAACATGCCTCAATGGAATCAGGTGGGGACAATATCATCCACCGCTCCACCTGCAGGACTACAGCAGGGTTTCCTTATCCCATCAGGAGCCCACCAAACCGGAAATAACTGCGGATCAAGCAATCTGCGCACCACTCAGGCAAGC**TGA**GACAGCTGGCGAAGGGTAAAGCTGTGCCTCATGGGGTTTAGATTATCGGGCCTAGTTCTTAAAGTGGACACACTTTATCTGACAGTTTACAAAAAACTTTCAGGTCCATAAAATGTGACTCGGGTTATTTGAATCTGATCTAACCTGCTATTTAAAAGACTGAATGTGtACTTTTGATAGAGGCACGAATGGGT

***wnk1b* in situ primer(1008bp)**

*wnk1b-*SP6 forward: **^5’^**ATTTAGGTGACACTATAGAAGTG**GGCAACGGTCCAGCACAGAG^3’^**

*wnk1b-*T7 reverse: **^5’^**TAATACGACTCACTATAGGGAGA**TAAAGGGGTGGGCACCTGGG^3’^**

(reverse complementary: **^5’^CCCAGGTGCCCACCCCTTTA^3’^)**

***wnk1b* QPCR primer (209 bp)**

*wnk1b*-QPCR-forward: **^5’^**CCGGGTCAGCTGTGCCCAAG**^3’^**

*wnk1b*-QPCR-reverse: **^5’^**TGGCCCAGGGGTTGTGAGGT**^3’^**

(reverse complementary: **^5’^**ACCTCACAACCCCTGGGCCA**^3’^**)

***wnk1b* Up MO: ^5’^TGCGTAAATTTCCTGCTCTTGCTT^3’^**

(reverse complementary: **^5’^AAGCAAGAGCAGGAAATTTACGCA^3’^)**

***wnk1b* ATG MO: ^5’^TGGGATTTTCCGATGACATCTTTCC^3’^**

(reverse complementary: **^5’^GGAAAGATGTCATCGGAAAATCCCA^3’^)**

>*wnk1b* contiq

AACTCTGTGGTTCACGTGAGCGTTTATTTTTTTCTCAATCTTTTTGTGCCACAGTTGA**AAGCAAGAGCAGGAAATTTACGCA**AAAGGTCAAAGTTTACATTTACGCTTTTTTCCAAAATTATTTATTTATACGTTCAAGTTTATTTTTCCCT**GGAAAGATGTCATCGGAAAATCCCA**ACAAGGTGGTGACCTTCTTGGCCCCGCCACCTCCAAAGAATGTGAATGGCTCCGGTTCAGACTCGCTGGTTGGTGAGAAGCTAGACACAGAGGTCAGAAAGCGACGCCACACTATGGATAAAGATCTGAAGACGGCCGAGCATAGATTTTTCAGACGTAGCGTCATCTGTGACTCCAATGCCACAGCCCTTGACTTGCCCAGTAAGGCCTGCATCCTCACCTCACCTCCAGACTGCGAGCAGTTTGTTATACCCACGGTGCTGAGTTCGACCCCTGATGTAACTCTTGACACCCAAACAGTAGTTTCGGCGGCCGTAGTGGAAGAACGTTCAGAGGATGAAAGACGTGCTGGGGTTGAAGGGATTGTGGAGGTACAGAGTCCTTCTGTGCAGGATACAGGCTCAAGGCAGAGTGAAGTCCTTGGGAAGGTAGAGACCGAGCTCAGTGATGTTGGTGACAAAGAGCAAGAGAGTCGAGTGACAGACAAGCAGGAGGAGGAGGAGAGAGGGGTGGCGAAGGCCCGTGCTGAAGCTGAGCTGAGGGAGGCTGTGAAAAAGGAGCAGGACGAAATTGAAGAAGTCGAGACCAAAGCTGTGGGCACCTCTCCTGATGGCCGCTTCCTCAAATTTGACATCGAAATTGGACGTGGATCATTCAAAACGGTCTACAAGGGCTTGGACACAGAGACAACAGTGGAGGTGGCGTGGTGTGAACTTCAGGATCGGAAGCTGTCCAGATCAGAGCGCCAGCGATTCAAGGAAGAGGCCGGCATGCTGAAAGGCCTGCAGCACCCAAACATTGTGCGTTTTTACGATTCATGGGAGTCTCCTTCCAAAGGGAGGAAGTGCATAGTGCTGGTGACGGAGCTAATGACATCAGGAACCCTGAAAACATATCTGAAACGCTTCAAGGTGATGAAGATAAAGGTGTTGCGAAGCTGGTGCCGACAAATCCTGAAGGGCCTGCACTTCCTGCACACCAGGGCTCCACCAATCATCCACCGGGACCTGAAGTGTGACAACATCTTCATCACCGGACCCACCGGCTCAGTAAAGATCGGTGATCTGGGCCTGGCCACCCTCAAGCGTTCTTCGTTCGCTAAAAGCGTCATAGGTACCCCTGAGTTCATGGCGCCTGAGATGTACGAGGAAAAATACGACGAGTCGGTGGACGTGTACGCCTTCGGGATGTGCATGCTGGAGATGGCTACCTCAGAGTACCCGTACTCCGAGTGTCAAAACCCTGCTCAAATCTATCGCAGAGTCACCAGCGGAGTTAAACCTGGCAGTTTCGACAAGGTAGCCATTCCAGAAGTGAAAGAGATCATCGAGGGATGTATTCGGCAAAACAAAGATGAAAGATACGCCATTAAAGACCTTCTGAACCATGCCTTCTTCCAAGAGGAGACCGGGGTGCGGGTGGAACTGGCTGAGGAGGACGATGGGGAGATGATCGCCATCAAGCTCTGGTTGCGCATTGAAGACGTCAAGAAACTGAAAGGCAAATACAAAGACAACGAAGCCATCGAGTTCTCCTTCGACCTCCATCGGGACGTGCCAGACGATGTGGCTCAGGAAATGGTCGAGTCGGGTTATGTTTGCGAGGGTGACCACAAAACCATGGCCAAGGCCATCAGGGACCGTGTGTCTCTGATCTCCCGCAAACGAGAACAACGACAACAAGTGCGAGCCGAGCAGGAAAAACGCAAGCAGGAGGAAGAGCAGAAACTGTTATCCAGTGAGTCTATTAAGAACGTGGCCGGAGCTCAAGGAACGCAAGGTTCTCTACAGAGTCAGTGTAGCTCCCAACCTCCAACTCCCGGCCTTGTGCAGCCCGAGTGTGAGGAGCCTGATGCAGATCCGCAACATCAATACATGCAAAGTGGAATAACCTTGGCGGACGGGACATTTGACAGTGGTCAGGGTTCATCAGTGTTCTCAGAACCTCACCTCAGCCAGCTAAGCATGTCGTATAGTTCTCCTGGAACATCCCAACAACAGCAGGTCCCAGGACAGGGCACCTACACTCCCAGCTCTCAAGCTCCTCAACAGCACACCGCATATCCCCAGCAGCCCATGGTTCAAGTCCCCATTCCTCAGTCTACAAGTGGCATGTCTCTGGCTTCCTGCAGCACCCCAGCCGCCTCTGCCTCCCAACAGTATGGAGTTTACTACGTCCAAGCACTCCCTACGCAGGCTCCTGTTCAGCAGCCGGCCTCTCTAGGTCCCACTTCCCAACCCTGTGCCTCGCTACCCCAACAACAAACACCCCAACAGCCTTCCAGCACTGTAGCTCAGAGCATCTCCCAAACGCAAATCACGCCAACACAGACGTCTCACAGTGCATCGCAGACTTTGACAACAGAGCTGTCACAACTCCAAACTCCTGCTGTGGAGAGCAGTCATTCTGATGTAGCTTCTGGGCTCAGTGATGGCAATGAGGGCGTAACAGGTGGTAGACATGAGGGTCGTCCTGCCAAAAGACACCAGCGCAGATCGGTGCGCAGCCGTTCACGCAATGAAAAGACCTCGAAGGTCAAGCTCAGTGTGCTTAATATCTCCAGCATGGGTGACAGAGTTGCAGAGTGTCAATTAGAAACACACAACAGGAAAATGGTGACTTTCAAGTTCGATCTTGATGGTGATAACCCAGAGGAAATTGCACAGATAATGGTACAAAGTGAATTCATTCTTGAAAGCGAGCGCGAATCGTTCATCGATCGGATCCGAGAAGTGATCGAAACAGCTGATGAGAAGGGGGTGGAGAGAGAGCGCAGCCAGATGGCCAGTGATCATGAGCAGCTTATGCCCACAACATCTGCGCCTCTTGAACACGGTGTTCCACCTAGTTCAGCTGCACAGGTGGTTCATTCTGCAGGCCGCAGGTTTATCGTGAGTCCAGTGCCTGAATCCAGACTGAAGGATCAGTTCTTTAGCCCATCCCAACCTACAATCCCACTTCATGAAGAACCTGCCCCTGTGCCAGTTGCATTGCCTGCCTTGGGGCTCTCTGTGTCTGCGTCTGCTGTCAGCCTGCAGCAGGCGTTCACTGAGATGCGTCAAGCCCACTTTGACCCAGGTCCCAGCACTGCACCCCCTATGCTCCACAACTCCATGCCCCCTCTGCTTCCTGCCACAACACCCTTGCCCTCTAGTGCCACCCCGGTGGTCTCTCCCCCAGTGTCTTCAGAAGTGACCTCTTCGCCATCTCTACCTTCTATAAACCCTCCAGTGTCATCATCTCCTCCTCCAACCGTCCCGGTCAATTCCGTAACCTCCCCTCCAGCACAACTCACCCTGCCTCAAAGTCAAGTTGCGCCTGCCGTCATCAGTGTTATTCCCACCCCTTCCACTCTGCCAATGCCAGCGGTGCCAACTGCCACATCCTTTCCACAAACTTCAGTCACCGTACCTTCCAGCACACCAATCGCTCCAATGGGATCAGTGTCCGGAGGCAGTGGAGAACAACCATCCATCCCTGCTACTTCTTCGCCCACTAGCGCTACAATCCAAACGCCACAGCTGGCACCATCCACCATCCCCACCACCACGGCTCAGTCACAAGTGCCCCCTCTCCAGCCGGTGACCACAACCATGCCAGTCGTGCAGCCCACACCAGTGCACAGCCAACCACAGACCACCACCGTGCCCAATCAGAGCCATGCGCATAGTGTTGAATGCGACAGTGACCAGCAGGGCAAGGTTGATGACATCCAGGCTCTAGATAAGAAGCTGCGCTCGCTCTTTATGGACTTGGGCTCCGGTCCCCCATCTGCCCAGTCTGATGTCACATCCGACCCGACGGCGACTCCAAGCGTTCCTGGCACCTCGTCTCCAACCACATGTGCCACCCCCAGCGGGACTCCACTCCCTCCCTCTAGTTTACCCCTGAACTCCTCCACTCAGTCTGCAGGGTCTCCCATGACCTCCATGGGCAATGCCTCCACCCCGGTCGGCTACAGTCAGACCACTCCATCCAAAGCTCCCTTAACACGATTACCGGTGTTGCCTGTCGGCCCAGATCAGGCTGGCACGCCGCCCACAGAGCACCTTCCACCCTTCCCAGGACCTGGTTTAATTCAGTCCCAGCAGCCCCTGGAGGATCTGGATGCCCAGCTGAGACGGGCACTGAGTCCGGAGACCGTCCCAATCAGCAGTGTCACGCACCAGTCATCTCTAACCGGGATGCCACCTGGAGGACAGCCAGTTCCGTTTTCTCTGGATGATGAGCAAGCAGCCGCACCTGCAGCTGGAGGATACAAACTGGGGCGATTCCAGGTGTCGGTGGCCACTGATGAGAACACCACACAGACTCCAACCAGCTCATCCTCTTCCACATCATCTTCCTCCACCACCTCCTCTTCCTCCTCCTCCTGTAGTCTGTCCAGCCCTGAAAACACACTGCACAGAACCTCCTCTCCCCTCATAGACACAGGCGCAGATGTGGTCGATGGTCTCCCTGCTCTCCCCAACCAGACAACGACCCAGCCTCAGCCCACCACCATCGGCCGCTTCCAGGTCACGACCAAAACAGACACGCAGGTGGGACGCTTCTCTGTTAGCAAAGCACAGGACGAGGTGACTACATCCACCTTACAACCTCAGCCCACACCTCAAACCCATTCTGCTGTG**GGCAACGGTCCAGCACAGAG**TCCCGGGAGCTTAAACAACTCCATCAGCTCCTATTTCAGCAGCGATAATGACTCGGAGTTTGAAGACGAGGACTTCAAGAGAGAAGTCAGCAGGCTGAGAGACAAGCACATGATAGAGAGTCAGGCTCTATATTCTCGTCAGAAAGCAGAGATTGAGGCTCTGTTTGCCCGTTTGGGGAAAGTTCCACCTGCTATGGTTAACCCACCTGTGGTGAACCCAGCCGGCCGCAGGAGACGCCCCACCAAGAGCAAAAGCAGCAAATCCAGCCGCGGGAGCTCGCAGGCTAGCAAGAGTCCCGTACAGCCAGCAACCAGCACTTTATCAGCACAGAGCGCCCCCTCTGTGTACCCAGCCCAGCAGGCTTTCCTGGCCCCCGGGGGGATGATGGATGGGGGGAGCAGCCCTCTGCTGCAGTCTTTCAAACCCTCGCCCTCCAATGACAACCTGTGCTCCACTTTCACCAGTGACGCCACGCTCTCTGCGCCCAGTCTCTGTGTAACCTCGCAAGGGACGAGCAGCACCAACACGGTGGCAGGACCCGGTCAGAACCAAAGCCAGCCACCCGTCTCTCAGTCTCAGAGCTGCAAGGGCATGTTCACAGACGAGCTGCACAAACTGGTGGACAACTGGGCCCGGGACGCCATGAAACTATCACAGGGCAAGAGAGGCTCCAAACACCAACAGCAAGTGGTTCCTCAGGGCCACAGCTACGAAATGGTTCCTCCAGTAAACATGGGCCGTAAGTACTCAGCTCCGGGTCAGCTGTGCCCAAGCATCGCTTCAACTCTGAGCGGCCATCCACCAATGCCCAACACTCCCGCTACCTCTCTAGGAGCTCGGAAGGGCTCCTTGTGTCCCACGCCGCAGTACGGCTACCCCTCCGCACCTTACAGCGCCCAGTGGGCTGGAGCGGCCACGCACACCCAGGCCGGTCTGCTGGCCACCTCACAACCCCTGGGCCAATATC**CCCAGGTGCCCACCCCTTTA**CAGACCTTCCACATCAGCACCTTGCAAAAGTCAGTCAGCCACCCGGGTGGACCCAATCTAAAGAGCACG**TAG**GGCAGGGCCTAGCCGCGCCAGGCCTATAATAACTGTTGGGGATTAAAGCAAGGGTAGGGGTGCAGACTGAAAGTGGGTCTGGGCAAGCATCCTGCATGCTCACAAATATGCCACGTACGTAATATAAGCTTGGAATTCGCCCGGGTACCGAGCTCGCCCTATAGTGAGTCGTATAC

***flt4* in situ primer design (720bp)**

*flt4*-T3-forward: **^5’^**AATTAACCCTCACTAAAGGGAGA**CTCCATCAGATATCAGGGATCCT^3’^**

*flt4*-T7-reverse: **^5’^**TAATACGACTCACTATAGGGAGA**TTGGGTTTTAGGAGAAC^3’^**

(Reverse complementary: **^5’^GTTCTCCTAAAACCCAA^3’^**)

*flt4* MO: ^5’^**CTCTTCATTTCCAGGTTTCAAGTCC**^3’^

(reverse complementary: ^5’^**GGACTTGAAACCTGGAAATGAAGAG**^3’^

Against *flt4* translation start codon, -17 to +8.)

>gi|56548633|gb|AY833404.1| Danio rerio Flt4 (flt4) mRNA, complete cds

ACGCGGGGTCTGTTGGCTTTATTAAATGACATCAGAAAACGCGCAGGTTTGCTAGAATATCTAAAACCCCGGA**GGACTTGAAACCTGGAAATGAAGAG**AGATTTTACGTTTTTCTGTCGGATTTGGATTGGGATTCCCTTCTTCTCAGGTCTGGTGAATGGGTTTTCTATGAGTCCACCCACCCTTGACAACACCAAAGACCAGCTTGTGATTAACGCTAACGACACACTGAACATTACATGCAGGGGTCAGCGAATTCTGGACTGGAGCTGGCCTGAAGAGTCTTTGAGTAAAGTGGAGTTTACTGATCGCCAGGGTCAGCAGTCACCCACTGACACCCCAGGGTATAGAGAAATCAGGCTGAAGGAGTGTCAAGGGGTGGCTGGAAAACCCTACTGCAAGATCTTAATACTTACCAATGCCCAGGCCAATGACTCGGGTTATTACCGCTGCTTCTACAAGGACATTAAAGCTGTTATTGATGGCACCACGGCAGCCAGCATTTTTGTGTTTGTTCGAGACCCAGAGCATCCATTCATCAAAAGGGGAGACAACGACATGGAGACCATCTTCATAACAGACTCTGAAACACACATCGAAGTACCATGTCTGGTTTCAGATCCTGACCTAAAAGTCACTCTCTTCTCGTTAGTGCCGTATCCAGAGCCTGTGGATGGCAGTGTGGTCACCTGGAATAATAAAAAGGGTTGGTCGATTCCCAGGCATATCATTCAGAACACTTCCACCTTTATTGGCTTCTACTGCTCCATCTCAGTCCAAAACAGCCAGCACACATCATCAATCTATGTTGTCCAGGTCATTGGGCTGAAGTTTTATGAGTTCAAGCTGTTTCCTGAAGACTCGCCTGTGGAGTTGATGCAGGGGGAGAGTTTGGTGCTCAACTGCACTGCACTGGTCGACTTCAACACTGGTGTGGATTTCCAGTGGGACTATCCAGGCAAAAAGGAGAACCGGTTGGCGAGTTTACAGCCTCTGCGTAACGTCCTGGATGAAGCCACGGAAATCTCCAGTATCCTCTCAATCAGAAACATTCACTTGGATGATAGTGGCTACTACACCTGCTGGGCTAATACTCTGGAAATGAAACGAGAGCTCACTACTGTAGTCATAGTTCATGAAAAACCTTTTATCAGCTTGGACTACAGGAATGGATCAGTTATAGAGGCAAAAGAGGGACAAAAATCTGTCCGGCTATCCGTAAAAGTGTCAGCATACCCTTCACCAGAAATACAATGGTATAAGAATGGAAAACTGATCAGCAGCAAGAATTCAAGCAGGTTTAAGGTCCAGCAGCACTCTCTACAGATCAGGGATGTGTGTAAGCAGGATGCTGGAGAATACATGCTGGTCCTGAAAAATAGCCCTGCAGCCCTGGAGAAAAGACTCAACTTTACTCTCATAGTCAACGTTCCACCTCAGATTCATGAGAAGGAGGCAGCACCACCTACAAACCTCTATGGGAAGGGCACTCGACAGATCCTCACTTGCACCGCAGACGGGAGCCCTCCAGCCTCCATCAGCTGGCAGTGGAGGCCCTGGAGCCCCTGCGATCTGGAGCGTACCCGCAGAGCCCTGAGACGGAGGGGTGGACGAGACCAGAGTCCTTTCTGTCACAACTGGATGGATTTGGACCCTGAACATGCTGTAAACCCTATTGAGAGTATTGACACATTGACTCAGATGGTGGACGGCAAAGAAAAGACTGTCGGCCGTGTGGTTATTCAGAATGCCAGTGTGCCAGCTATGTACAAGTGCTTGGCTGAGAACAGAGTGGGAAAAGATGAACGACTGATTTATTTCTACGTAACCACTATCCCTGAAGGATTCGATATAGAGATGGAGCCCTCAGAGGATCCGCTTGAGCAGGACCTGGTACAGCTGAAGTGTAATGCAGATAATTTCACCTATGAGAACCTGCGGTGGTACCGCCTGGACCCGCAGACTGTTCCTCCAGAGCTGGACTGCAAGAGTCTGCACCAGTATGCCACATTTTTGGAGGGACAACTATCTTTTCAGACCACCAGCAACAACTGGGTCCTGCAGCTTAACATTACCAACATTCAGCTACAGGATGAGGGGAACTATGTGTGTGAGGTGCAGAACCGACGAACCGGGGTGAAACATTGTCACCGCAAATACATCCCAGTCAAAGCAATGGAAGCACCTCGATACCGTCACAACCCAACTAATCATACGGTAAATGTGAGCGAGTCGCTGCAGATGAACTGTGATGTGGAAGGCACACCCTTTCCTCAGCTGTCCTGGTTTAAAGATAACCAACCC**CTCCATCAGATATCAGGGATCCT**CCTTCAGGACTCCAATCGGACTCTCAGCATTCAGCGAGTGCGAGAGGAAGACGCCGGTCTGTACACCTGCTCTGCCTGCAACCAGAAGGGCTGCGTTCAGTCCTCCGCCACGGTGTCAGTGATTGGCTCTGATGACAAAACCAATGTAGAAATAGTGATCCTCATCGGCACAGGAGTCATCGCCATCTTTTTCTGGGTCCTTCTTCTTGTCATCTTCTGCAATGTCAAGCGAGTGAACCCAGCTGATATCAAGACAGGCTATCTGTCMATCATCATGGACCCGGGGGAAGTGCCGCTGGAGGAGCAGTGTGAATACCTGCCRTACGACTCCAGCCAGTGGGAGATTTCCAGAGACAGACTGCGCCTCGGTAAGGTGCTCGGCCATGGAGCTTTCGGGAAGGTGATTGAAGCCTCAATCTTTGGTCATGACAAGAAATCCTCTGCAAACACAGTGGCTGTCAAGATGTTGAAAGAGGGAGCAACAGCCAGCGAGCACAAAGCCCTGATGTCAGAGCTGAAGATCCTCATTCATATTGGAAACCATCTCAATGTTGTCAACCTTCTTGGAGCTTGTACCAAACCTAACGGTCCTCTCATGGTTATAGTGGAGTATTGTAAATATGGAAATCTGTCCAACTTCCTGCGTGCCAAGAGGGAGTTTTTTTTACCATACAGGGATC**GTTCTCCTAAAACCCAA**AGTCAAGTGCGACGGATGATAGAAGCAGGTCAGGCGAGCCAGAGTGAGCATCAGCCGTCCACTTCCTCCACCAACCCTCCACGTGTTACAGTGGATGATCTCTGGAAAACTCCACTCACAATAGAGGATCTGATATGCTACAGTTTTCAAGTTGCACGAGGAATGGAGTTTCTGGCATCTCGTAAGTGTATTCATCGAGATCTGGCAGCCCGAAACATCCTCCTGTCCGAGAACAATGTGGTGAAGATCTGTGACTTTGGTTTAGCACGGGACATCTATAAAGACCCTGACTATGTGCGCAAAGGCAATGCCAGGCTGCCGCTGAAGTGGATGGCTCCAGAGAGCATCTTTGATAAGGTTTACACCAGTCAGAGTGACGTCTGGTCTTTTGGAGTTCTGCTCTGGGAGATTTTCTCACTAGGAGCGTCTCCATACCCCGGCATCCAGATTGATGAAGATTTCTGCAAACGACTCAAAGACGGTACCAGGATGAGAGCACCAGACAATGCGTCCCCTGAAATATATGGCATCATGTTGGCCTGCTGGCAGGGCGAGCCCAGGGAGAGACCCACATTTCCAGCTCTGGTGGAGATACTTGGAGATCTACTGCAGGAAAACAGTCTACCAGAAATCCCGTTCAATGTGTCTCAGAGCTCAGAGGACGATGGCTTCTCACAAGCTTCTTCCAGACCACCATCACAAGAGGAGATCAGATTGGCCTGCAACACACTGCCAACACGATATTACAACTGCGTGCCATTTGCTGGCTGTGTGATGGTCGGGCCCTCCAGCACATGTCATTCTCGAGTGAAGACCTTTGAGGAACTTCCTATGGAAATGACATCACACAAGACACAGCATGACAGTCAGACAGACAGTGGGATGGTTTTGGCATCAGATGAACTGGAGAGGTTTGAACACAAGCACAGAGGAGCCATGTTGACAACTGCGACGACGGGCCAAAGCACTGATCGCCTCATCAGTTGTCCATCGGTGAGCAGCAGCGGCAGCGGTGGTGGTTTGCTCCGTCCAGTGTTCTTCACTCAACTCTCAGGCCAGACCTTCTACAACAACGAATACGGTCATCTGTCAGAGGAGGGCGTCAGCGACTACTTCTCCTCCTCAGACCAGGCCGTTTAATAAGGAAACCTGCCCGGCAACCTCCAACCTGTAACAAGACTGATCGGGCAGTCATATCCCGGTCCCGACCCCCCTTCTCCCACTCACACTTACTGAACTGACTGTAGCTTTGTAAAGTTCTCTCAGCAGCCACTGGGTGGCACCATTACATCAATTTCAAAACCAATAAAGTATTTTATCACTTTAGTTCCATATATCTCTATATAAAGAGTAAACAGCTTTTATTTGTTCAAAAAAAATGAGAAATGAAGCAGAATGGAGAAAAAAAAATAAAGGAATATATCTTAAAAAAAAAAAAAAAAAAAAAAA

***vefgc* in situ primer design (459bp)**

*vefgc*-T3-forward: **^5’^**AATTAACCCTCACTAAAGGGAGA**ATGCCATGCAGGAGCATTCA^3’^**

*vegfc*-T7-reverse: **^5’^**TAATACGACTCACTATAGGGAGA**TGGTGACCGGTTTGGTCCCT^3’^**

(Reverse complementary: **^5’^AGGGACCAAACCGGTCACCA^3’^**

>gi|45387874|ref|NM_205734.1| Danio rerio vascular endothelial growth factor c (vegfc), mRNA ( 537- 995)

**ATGCCATGCAGGAGCATTCA**GAGCCAGACCTGGTGGAGCAGTTGCGTTCAGCGGGTAGTGTGGATGAACTCATGAGGATAGTTTACCCTACCTACCGGATCATGCTGAAGTGCCGTTCCAAGATGGGGTCGCGCTTACTTCGCAGAGAACCAAGCTCCACAGAAACAAGGTCGGAGGAGGCCTCCTTTGCGGCTGCTTTCATCAATCTTGAACTTTTGAAAAGTATTGAGATAGAGTGGAGAAAGACGCTGTGCATGCCACGCCAAGTTTGTTTAGATGTGGGGAAAGAGTTTGGGGCTACAAACACCTTCTATAAACCACCCTGCGTGTCTGTCTACAGATGTGGGGGCTGCTGTAACAGTGAGGAGCTTCAGTGCAGGAACATCAGCACTTCATACATCAGCAAGACGTTGTTTGAAATCACAGTTCCAGTCAAGCA**AGGGACCAAACCGGTCACCA**

**Etv2 in situ primer design (517 bp)**

*etv2*-T3-Forward: **^5’^**AATTAACCCTCACTAAAGGGAGA**tcgtccctctctgcactcca^3’^**

*etv2*-T7-Reverse: **^5’^**TAATACGACTCACTATAGGGAGA**ttacactggccccaccgctt^3’^**

(reverse complementary: **^5’^aagcggtggggccagtgtaa^3’^)**

>etv2-NM_001037375

acgcgtccggcactgtcaaaacccctgatatagtgaaataaggactcagtgaaaactttaagat**atg**gaaatgtaccaatctggattttacacagaagacttcagaactcaggaggttcctgctggtttcgacttcagttcatatgactgcagtggtgaagacctgtcctttttattagacagcaaaggacccgtccaacagcagtatgccgaaaactactccgagcctcagaaggagctgttgcacaaaggtcatgttctcacagtcgactctgggctgttcaatctggactctttccctgagttcagcaactgggcagcatacactaatattccagaaggaatggttgcagacagacagcaggttggctttcaggaatcaactcaaacctaccagaacc**tcgtccctctctgcactcca**gcacaaagcagtacattcagtcccacaatggacaccagcagccattaccaacctgggaaaggcccaagtcacagaggagcatccgggaccgccagccttgaccatttgggtgaatcagacagaacatatggtttgtatgaagcagagcagcaaagcaggtcttcatattggtccgactacccctcacccggttactgcagctccatgccgctgagccagcctgcatcctcctcatcccctccggtcagtcagtccgctgagcacttctgcccccgcgtggtcaaaagacgcagcgctcctcctcaaagatcagacagagagggcgagatcacgccaatgtccgcctacccaggatctggacccatccagctttggcagtttctgctagaactcctgctggattctgcttgccacacttttataagttggactggtgatggctgggagtttaaaatgtcagatcccgctgaggtggcg**aagcggtggggccagtgtaa**aaacaagcctaaaatgaactatgagaagctcagtcgtggcctgcgttactactaccacaaaaacatcattcacaaaacggcgggaaagcgctacgtctaccgctttgtctgtgacgtgcagggcatgcttggaaaaactgcacacgaagtcttagcaagtctaaacatctccccgaatgcagcatctccacagtctgtagcaaacacaagccgctcagaggaaaccacagagtcctggacacat**tag**aggaggaattctcgaaggattggaaagaactttaaaacatcagacgtcgtgcctttggaggaagaaagaactgactgacttgagatttatgtactttaatttggactttgcagcagattgccaacaacagatctgaagtcagctttgaaatcaagctaggtgcctttagaaaaccatgttagctacctcttttcacattggaacattaatcttgtaaatgtattttgttttatcttatgtaaactgatggtttgacatcagatagtgaactcttgt

*flk1* MO: ^5’^**GTCTGTTAAAATAACGTCCCGAATG**^3’^

(reverse complementary: ^5’^**CATTCGGGACGTTATTTTAACAGAC**^3’^)

Against *flk1* upstream of translation start codon, -28 to -4.)

>Danio rerio flk1,vegfr2, mRNA, complete cds. mRNA sequence: [AF487829](http://genome.ucsc.edu/cgi-bin/hgc?hgsid=100330224&g=htcDisplayMrna&i=AF487829&c=chr14&l=37408691&r=37476122&o=all_mrna&table=all_mrna) Position: [chr14:37408692-37476122](http://genome.ucsc.edu/cgi-bin/hgTracks?hgsid=100330224&db=danRer5&position=chr14%3A37408692-37476122)

AGATAAATGGCTGTTACACCTTCACTACATAGTGTAGTTTTGGAAATTTCATTCATTCGGTTTTAGGAAGGACGGACAGCGGTCGCACCTGCCCTCGGGAGTTGTGGAGTCAGTGGAGTTTCTCCATTCGTCTTAGTACCGGGGCAGGCGCAGTCCTGCGCGATACTTTACAGTCACTGAGAGGTGTTTAACTATTAACACGTCACCGAAGAACCATCTCTATCATATCAACAGGAACTATAAGCATATTATCATACATCAAGAGAAATTAAGTTCTGGACTACTGCAGCCGCATCCGAACGTGAAGTGACATACGGAGTAT**CATTCGGGACGTTATTTTAACAGAC**AAAATGACTCCTCTTAAAACCTCAGTCAAAGCCTTCTTCACTCTTCACGTGCTTTTTAGTTGTATTTCACATGGTTTGGTAGAGGGATCTCGTCTGCCTGATCCACAACTGCTTCCTGATGGAGATACACACCTTCAGCATGTTGGTGGGACACTCACACTCATTTGCAGAGGATCAACTGCACTGCACTGGAGGCTGGCTAGCAGAAATGTGTCATCGGTACGCATTGAGTCGTGTGAGGAGAGACTGCACAAGCACTGCAGCAAGCTAGTCATACACAACTTGAGGCACAACGACACTGGCATCTACTCATGCAGTCACAAGAAGTCCAGCGATCATGAAGTGTCCACATATGTTTTTGTTAAAGATCCCCATCATCCATTTGTGGAGGCTTACAGTTTACCACATCCGCTTTTTGCATATCGAAATGATCCATATTTTGTGGTCCCCTGTAGAACAACATACCCAAACCAAAACGTTATCCTTGAGACGCAGATGAATCCTATGGCAGATGATGTTAAAAGAGGGGTACAGTGGGATCCAAAAAAAGGTTTCACGGTCCCTTTAAAACCTTATGACAGCTACCATCTAATTACATGCTTGACCAGGGTGGATAACGCAGAATTTTCATCAGTCTACCTACTTAAAAGGCTAACAATGGAAATTAAAAACCTTGCCATCGAACCAGAAAGACCAAGAGTACTTGTCGGTGACACCCTCATCCTCAACTGCTCTGCAGAGACCACTTACAATGGCAGGATTCACTTTGAGTGGGAGTTTCATAAGGAGCGGATCAATCGTaCTCACCATTTTAGCACCACACCGGTCCAACTTGCCCAGATTATGGTGATGTCTAAAGCATTAATCGTGCCTAATGTGACAATGGAGGACAAGGGCACATACACGTGCACAGGATCAATTGAATTTAAAAAGCTTCAAATGTCAACCAAAGTTATTGTTTATGAACATCCATTCCTCAATGTTACTCATAATAAACGTAAATTCACCTCTACTGTAGAAGGTAGAAGGGTGCAGTTTGAGCCTCGGGTCAATGCTGTTCCTGCGCCCGACAGAGTCTTGTGGTACAAAGATGGAGTGGCCATTTCTGAAAATTCCACATGTTACGAAACTGCAGGCTACAACCTGACCATAAAACAAGTGAGGCAGAAGGATGCTGGGATTTTCACTATCGCTCTGTCAAACCAGGAGAGGGGTCTCTACAGAAACATCAGTTACAAGCTTGAAGTCAGAGTGAAGCCAAAGATCTTTGAAGAGGATGTGGCTCCAGCAGGTCCCCAGACCTTCAGATATGACCAGAGGCACAAGCTCACCTGCACTGCATTTGGTATTCCCATGCCGAACATTACCTGGTTTTGGCAGCCATGTGACCCCAGTGCTAATCTTACAGAATGCAAACTTTACACTGATCCTCTTCCCATTGAAAACGTTGATGAccaTTTTCCTCAAAATCCGATAAAGGATGTAAACAGCAAGGTTGGATTGTTAAAAAGTAAAAACCGGACTATAAGTACCCTGGTGGTGAAAACAGCTAATGTGTCTGGGGTCTACTCCTGTACAGCAAGGAATGAACTTGGCAACCGGACCATGAGAATCCCTTTTTATGTGGATGATCACCCACAGCCTTTTGAGATTGAGCCCTCCACTGCGGTTGCTGGAGATGACATTACATTGACTTGCAGAGGCACAAGATACCTCTACGATAGGCTGACTTGGTATGATCCTCTGGGCcATAAGGTGCCCAAAGATGAAACCACTCTGCGGATTGAGCCCTACACTATATCTTTGTCAATCAAGCTGCCCAATGTTTCCAGAAACCATACACTCGGCTATGAGTGCCAGGCTTTAAAaATCAACACCAATAAAGTGGTCAATGTAACATCTGCTTTGACTATTGATGAGAGGCAAGGGCCCTGGTTAATGCAGAATCTGACCAATCAGGATGTAAACAGCAGCAGCACCCTAACACTGGCCTGCTTGGCGTATGGAGTTCCAGCACCCTTTATCACATGGTACAAAGACAAGACTCCTGTCACAGAGGGACCAGGAATCACTCTAAAAGATGATGGCACTCTAATTATTGAAAGGGTGAAGAAGGACGATGAGGGCATTTATGAATGTCGTGCTAGCAATGATGGAGGGGAGGCAAAAACCAGTGCGGTCATTACTGTAGTAGGAGAAGATGGTAAACCAAATATTGAGGTCATCATCTTGGTGTCGACTGGAGCAGCAGCAACATTCCTGTGGATTATGCTCATTCTCTTTATCCGCAAATTAAGgAAGCCAAGTTCAGCAGATTTGAAGACAGGGTACCTGTCCATCATCATGGATCCTGAACAGATGCCTCTCGATGAGCAGTGTGACCGTCTACCATATGACAGCAACAAATGGGAGTTTCCTCAAGATCGCCTCAGACTCGGTAAAACTTTGGGCCATGGAGCATTTGGAAAAGTTGTAGAGGCCTCTGCATTTGGCATTGACAAGATTTCAACATGCAAAACAGTGGCTGTGAAAATGCTGAAAGTGGGAGCAACAAATAACGAATGGAGAGCCTTAATGTCTGAACTGAAGATACTGATCCATATTGGGCATCATCTCAATGTGGTTAACCTGCTAGGAGCCTGCACAAAGCGTGGCGGCCCCCTAATGATtATCGTGGAATTCTGCAAGTATGGAAATCTTTCCAACTACCTGAGAAGTAAGAGAGGTGACTTTGTGGTTTACAAGTCTCAGGACGGTAAGGCTGTGCGTTCCAGCTCAGGCTGTGATCTGAGCGAGCTCATCAAGCGCAGGTTGGAGAGCGTGGCGAGCACCGGAAGTTCAGCCAGCTCCGGCTTCATTGAAGATAAGAGCTACTGCGACTCGGAGGAAGAGGAGGAAGAGCAAGAGGATTTGTAtAAGAAAGTGCTCACATTGGAAGACTTGATTTGCTACAGCTTTCAAGTGGCTAAAGGCATGGAGTTCTTGGCTTCGAGAAAATGTATCCACCGTGATCTGGCTGCACGTAACATCCTGCTGTCTGAAAACAATGTTGTGAAGATTTGCGATTTTGGACTTGCAAGAGATGTATACAAGGACCCAGACTATGTCCGCAAAGGAGACGCTAGACTTCCCTTAAAGTGGATGGCGCCAGAGGCCATTTTTGACAAGATCTATACTACTCAaAGTGACGTGTGGTCTTTTGGAGTGCTTATGTGGGAGATCTTCTCTCTTGGTGCCTCCCCTTACCCTGGCTTACACATTGATGAGGAATTCTGCTGCCGACTGAAGGAGGGCACTAGGATGAAAGCTCCTGAGTACTCCTCCTCTGAAATATATCAGACCATGTTGGACTGCTGGCATGGAGAACCATCTCAGAGGCCCACTTTCACAGAGCTGGTGGAGAGGCTAGGAGATTTGCTACAGGCTAGTGTGCAGCAGGAGGGAAAGCACTACATCCCGATCAACACGGCCCTCTTGACCAAAGCAGACCCCTCAAACCAGAGTCCCACAGAGGAGACCTCCACACGACCAGTCTCTCTCAGAGACTCTGGGACGGCTTGGAACATCAAGATCCGCCCAGAGAGTGTGAAGACCTTCGATGAAGTAATCCTGGAGAACGGAACCAACAAGATCCACGAGGGTGGGCAGTCAGACAGTGGGATAGGACTATCTTCAGATGACCTGAAGACGCTGAAGCGGCTGGAGTCCCTGGCCCGACCCCGGAGTTTCATGTCCCGAGCGATGAAGAGGAAAAGTAAGGAGTCAGTTCTGCTGGAGGGCGAAATGGACAAATACCCACCGCTTGTTCCCTCACTGAGTCTGGAGGACTCGTCCCTTGACTCGGAGATGGAGTGTCACAGTCCTCCTCCAGACTATAACTATGTGGTcCGTTACTCCACACCACCCGTCTGACATGCTCTGCGCTGGAAAAGCCGCCAGAAGTCTGATCGGTCCATTCTGTCTCATTCTGTCTCTGTCTCTACTTTTATTTTTCCCACACATGGTCATTCAGAGATTcACATTTCCTGTACACTTCTGTCAAGTTAGTGAAGGAaTACAGTCGGCTGCCACTCATATCTGTGGTAGTGTAAGATTCATATGGACCAGCTCAGGTATTTCTGCTGTCTACTGTGAACACAACAGGCACATTTTgTTACACTAACAGCACTGTTTCCCCATTGAAAATGCATTCTTCAGTCAGAATGGTGGCTAATTATGAAATCATTAGTTCGGTGGTTAGAGCGAGAGGACAAAGGCAATCCTTATTTTATTTCTAGATAAATATTTTATTGAAATGAAACTGGAATTTTGGTTATGTGATATTTTATTTTGCTAAACGTATTCTTTCAGAGCTCGCTAAAGCTTCTTCTTTGAAAGGGAAAAACTATTTTTCTTTAGAACAGATTTCACTCAAACTTTCAGACAAGCTGTCAAACAAAGACCACAGATCATGAATAATTTAACATGCGGTCAGAAGCTCCTTCCCAGAaCTCCATCACGGCACATTGATCATCAGCATACTTATGATGCACATTGTcTTTCCAGGTTCAGTCGCACCAGTCTGAGGGCTGATTTCCTGAGTCTTACTCGATAAAGCTAAAATGAATCACTTCTGTTCCCGCTTGTGCTATTTTTATCTGGTAAACATCTTATAATATGGTTAATTTAGTTAAAATTCAGTTTAAGATCTTCAACTTGCTTATGGATGCCACAACATTCAGTCCATCACTGTGAAAGTAAATTACTCATGaCAATTTCATAGGAAACATTCAGTTAATTATTGATCAAATGTGGATGATATAAAGGCCTTGCTCTGTTCAATACACAACAGTTACACTGCATCGTGTACTTTCCATATTTATTTCGTTTACACTACTGTATACATAACAGCACGTAATATGTTGTGCAGCTATTAACCAGAGGAGCGTATCCTGTTACTGATTCAGTAGTCGTTTGTGCTAAAATGCTACTAAAGCTTGCAGGGCAGGCTATGATATTGTCAACAGGCTGGTCACTATATTAATGTTCATGAAAATGATATGCACACATGATGTAGGTGCTATCAGTATTAACTCCTTAAtATCTGTATGTACGTAAGGGAAAGGTTGgGAGATTATCGTATTGGTTAAGCCAGATTATATTGACTGTGTAAAGGGCC

*pi3kc2α* MO: ^5’^**TATGTGGGCCATGGTGTCAGCTCT**^3’^

(reverse complementary: ^5’^**AGAGCTGACACCATGGCCCACATA**^3’^)

(Against *pi3kc2α* translation start codon, -12 to +12.)

>gi|528508131|ref|XM_005159306.1| PREDICTED: Danio rerio phosphoinositide-3-kinase, class 2, alpha polypeptide (pik3c2a), transcript variant X2, mRNA

TCGTACTTTATTCACACGCAAACTCTTTCTACGCGGTTAAGTTTATCCATCGGCTCTGCTCGTCCGTGTACCGGAAGTACAGTATGTTGTGTTTGTCTGAGTAGTTCTTCTGTTTGTTTTTCTTCGGCTTGGGTTTTCACTTTTTGTTGGCGCGTTCAGTGCGCCGAAATGCCGAAACATTTGCACAAACAGTGCCTAAAAGTTTCGAAAACAACTTTTTACGGGAGATATTTCGCGCACCTGTTGTAAAGTTCCCATCAGAACCCAGTGGTCTCCTCATGTACATCGCAAGGC**AGAGCTGACACCATGGCCCACATA**TCCAGTGGCAATGGGTTTAAGTTTGACAGGCCAGCCTCTCCGGGGGTGGTCCGGCCAAAAGGAATTGTGGGGAAGGAAGAGGCTTTACGCATGGAGGTAGAGGCCTTCGATAAAATTAAGCGCGAGAAAAGGCACACACTCCCAGTCACAGCCTCTTCTGTTAATCCCCTTCCCAATCCAACAGCGCAAACATCCAGCAGCCGGCCAGAGAAAGACCTTATTGTATTCTGCGAATCAGAAACACAGAAGAAGGAGCAAAAAGACAACTTTGAGGACATCGATTTGGAGAACCTGACAAAGGAGGAGCTGGAAAAGCTGCTTCTGGATGACAGTTTTGGTGCAAACAAAATGACAAGACCTTCCTCTTTGCTAGGTTGCAATCTCAGCGCATCATATCCTGGCGGACATGCCTTCAACCCCTCTTCGTTCCACTGGACACCCACTCCAACGCATGCACAGACTCCCATCTTCCCCTCTGCTCCATTCGCCAAGCCTCCTTGCTCTTTCCAGAATGGCTTTAGTCCAGCAATGTCTCCATTTATTCCACCGACCCCCTTTCTCTCCTTTACTCCCATCCAACCACCTGCTGCTCTGGTCTACTCGCAGCCTGCCGTCACCCCAGAGATGGCCAAGCTGTTTGACAAGATTGCCAGCACTTCGGAATACTTGAAGAACGGCAGGTCCTCTAGCATGGAAACCGAATCGGCAAGTGTCAAGTCTCTGGAGCCCCTACCTCTGCCCTCAGAACCTCCTAACATCAGCCGATTTGAATGGCTGGACTTGGACCCTCTCAACAAGCGCAAGGAGGTGGAGGTCGAGGAGACACCTGCTGTTTCAAGCTGTCCTTTTATTGAGGAGTCTGGGAAAGCTAAAGACCCGTGGGATGCGGTTCTTCAGGATGAGCATGAAACTGTAGATAATGGCAGTCCCACCGCAGAGGAAAAGAGCAAGGCAACTCAACCCAGAAGAGCATCCACAGGGGCGGCTGTAACAAAAAGCCAATCCCTCAGTATATCTGCAAACTCAACGAATCAAAGCACAGGCAAACAGGTCAATAAGGGAAGCACAAATGTACTGTCCAAGTACTCTGCCTTACAGGAGAAGGAAGCCCAGAATCTAGAAGTTGTAGCATTCTGTGAAGACATTTCAAAACTGAGGTCAAAGTTCCCACACGATGACCTGTCTACCAACCCTGGCTATGTTCTCAGCCCTGTGATCACGCAGAGGGATGCAGGAGGGGACAATGGGGGCAGTGTGAAAGTGTCCATTGAGATTTCTGATTCTCAACAGCCTGTAACGTTTACTTGCGATGTGACTTCTCCAGTGGACTTGCTCATAATGCAGGCTTTGTGTTGGGTTCATGATGACCTGAACCAGGTGGACATCAATAGCTACGTTCTCAAGGTCTGCGGACAGGAGGAGGTCCTTCAGAATAAACACAGTCTAGGCAGTCATGAGTACGTGCAGAACTGCAGGAAGTGGGAATCAGAGATCAAACTACAGCTCCTGTTTCTTAGCACAATGAGAAGAGATCTGGCCCGAACTGCAGAAGATGACATCTCGCCCATTGATTTGGAGAAATACCTTGGTCTAGTGGAGAGGCCATTTAAAGAGGCAGTCACAAGAGAAGGCCTTGCTGAATATCTAGAAGGCTTTCATAAGCAAGTCAATCTCTGCCTTCAGAATGAGAACAACCAATATAAAACAGTGGACAATGTGGTTCAGTCTGTTAAGAATCTGTGCTGTGCTCTGGATGAAGTTGAGACACCAGCCATCACTGATGCCGTCAAGAGGCTCAAGCGCTCTGTCAACCTTCCTAGAACACGCTCACCAGAGCACTATGCTGAATTGCAGGCTGGTGCCACATCTTCCGGAGGTTCAGCTAATGGTTACTCTAGCCCTGTAGAGGAGAGTTTAGCAGTGCTCACAGATGCTGTCTATGAACTCGCCAAGCTCTACCTTCAGTCTTTCTGCCCGATTAGCCCTGGTTTCATGATAGAAGAGCACAAAGAAGATGAGAGGGACAGCAAAGAGGCCTCGGGCACCACTGAACACTTACAGTTCACACTGTTTGCCGTTCATGGCATTCCTGCTACCTGGGTCAGCAGTTTTGAAAAATACTACCTGATGTGTGCCCTGACCCATAACAACAGGAATCTCTTCAAACCTGTCCAGTCCAAAAAAGTGGGAACATACAAGAGTTTCTTCTACCACATCAAATGGGATGAATTAATAAACTTCCCTATTTCAGTGTCGCTGTTACCACTGGAGGCTATGCTTAGTCTTTCTCTCTATGGGGTCCTCAGTCAGAATGCAAACAACTCACCAGATTCCAACAAGCAACGGAAAGGTCCAGAACTTTTGGGAAAAGTTTCCATGCCTCTGTTTGACTTCAGGAGGGTTCTGTCCAGAGGCAGTAAACTGCTGAGTTTGTGGACTTCTCCTCAAGCCCTTCAGCCTGGAGCTGCAGGCAAAGGGAGGAATCCTACAGAAAAGATCATACTGCAGGTTGACTTCCCCAGTCCAGCGGTGGACGTGCTCTATGTTGGTCCTCAGGAGAACGGCTGCCCCAATCCTCAGTCTCTAGACCCACTTGATCAAGACGATCGAAGTGAGATAGAAAAGCTGTGCGCACGAGCATCTACTTTCGGACTGTCACGGGCGGACAGACAGCTGCTTTGGGACCAGAGGTACTATTGTCGGGAATATGAATACAGTTTGCCGAAAATCCTGGCCAGCGCTCCCAGCTGGGACTGGGGCAGTATGGGGGAAATCCACGCACTTCTGCACCACTGGCCTGCTCTGTCACCCGTTTCAGCTCTTGAGCTCCTTGAATCCAAGTTTGCCGACACAGAGGTGAGGAAAGTGGCTGTGAGCTGGATCCAGAGCAGCAGTGATGATGAGCTGGCTGATTATCTCCCCCAGCTAGTGCAGGCAGTGAAGTTCGAGTGTCACCTTAACAATGCCTTGGTGAAGTTTCTGCTGTCTCGAGCTCTGGGAAATGTCAACATTGCACATTATCTTTACTGGCTGCTGAGGGATGCAGTGCAGGACCCGGCGTTTGGTCAGCGTTATGAGCGGATCCTCTGTGTGCTGCTGTGTCTGTGTGGGACCGGACTGAGGGCTGAGCTGGAGAAGCAGACCCGACTTGTGCAGCTGCTTGGAGCTCTGGCTGAAAAGGTTCGGCAGGCGAGCAGCTCAACCCGACAGGTGGTTCTCCTCGAGGGTCTAGAGAGGGTTCAGTCTTTCTTCCAGAAGAACAGCTGTCGACTCCCTATTAGCCCCAGTCTGGTGGCAAAAGAGCTCAATATCAAGGTCTGCTCCTTTTTCAATTCCAACGCTGTACCTCTAAAGATCGCACTGGTTAACGCAGATCCGCTGGGAGATGAGATTAACGTCATGTTTAAGGTTGGAGAAGACCTGCGGCAGGACATGTTGGCACTACAGATGATTCGGATTATGGATCGGATCTGGCTTCAGGAAGGACTCGACCTGCGTATTGTTAACTTTAAATGCATCTCTACTGGGAAAGATAAAGGCATGGTGGAGTTGGTACCATCTTCTGAAACGCTGAGAAAGATTCAGGTGGAGTATGGAGTGACGGGTTCCTTCAAGGACAAACCTCTTGCTGAGTGGCTTCGCAAGTATAACCCTGCTGAGGATGAGTATGAGAAGGCGTCTGAGAATTTTATCTACACCTGTGCTGGATGCTGTGTGGCCACTTACATCCTCGGCATCTGCGATCGTCACAACGACAACATCATGCTGCGCTCCACTGGCCACATGTTCCACATAGACTTCGGGAAGTTCCTGGGTCACGCACAAATGATTGGGAGTTTTAAAAGGGACCGAGCACCGTTTGTCTTAACCTCTGACATGGCTTATGTCATCAATGGAGGCGAAAGACCCACTAGTCGCTTTCAGCTGTTCGTTGACCTTTGCTCGCAAGCCTACAACCTGATCCGCAAACACTCCAACCTCTTCCTCAACCTGCTATCTCTGATGACACAATCAGGTTTACCCGAGCTGACCGGAGTCCAAGATTTGAAGTATGTGTATGATGCTCTTCAGCCTCAGACTACAGATGCAGAGGCTACCATTTTCTTCACAAGGTTGATTGAATCCAGTCTGGGCAGTGTGGCTACAAAGTTTAATTTCTTTATCCACAATCTGGCTCAGTTGCGTTTCTCTGGCCTTCCCTCCAATGATGAACCCATCCTGTCGTTTGCTCCTCGGACATACACTATGAAGCAAGACGGCAAGATTCGGGACGCCTCTGTGTTTTCATTTCAAAAGAGATATAACCCAGACAAGCACTATACGTATGTCATTCGGATTCTAAGGGAAGGTCAGAGCGAGCCACAGTTTGTTTTCCGCACATTTGATGAGTTTCAGGAATTGCACAACAAATTGACCATCCTTTTTCAGTTGTGGAAGCTGCCAGGGTTTCCCAGTAAGATGGTGCTGGGACGCACACACATCAAGGATGTGGCATCCAAGAGAAAGGTTGAATTGAATAGTTATGTGCACAATCTGATGAGGAGTTCAACAGAGGTCGCCCAGTGTGATCTGATCTACACCTTCTTCCACCCTATCGCAAGAGATGACAAGACTGAAGGGGTTGATACCCTATCAAAAACTCCAGACATGCCTCCAGTGAGTCCCACCACAGGCCGTGTGGAGGGTGAGGTGAAGCTGTCAGTGTCCTACAGAAACAGCACACTTTTCATCATGGTCATGCACATTAAAGACCTGATGTCCAATGACGGAGCAGACCCAAACCCTTATGTGAAAACATACCTTCTCCCAGACCCCCACAAAACTTCCAAACGCAAAACAAAGATTGCGAGGAAGACAAGGAACCCAACATTTAATGAAATGCTGGTGTATAGCGGTTACAGTAAGGAAACACTGAAGCAAAGGGAACTTCAACTCAGTGTGCTCAGTGCCGAGTCTCTGCGAGAGAACTGCTATTTGGGAGGAATAACCCTCTGCCTTAAAGACTTTGACCTCAGCCGAGAGACAGTCAAGTGGTACAAACTCACCACGGTGCCCTACTTCTAGATCCGGCCATCCCTCTACACCAGTCTGCCCTGAAAAGGTGACATTACTGCAACAAGTGCCCTTAAAGGACCACTCGCATACCCTCATAACCCCCTTCTCTTTCTCTATCTTTTTCTATGACACTAATCAAGGCTGAGTCCTTGTTGAAAGAATCAATCCACATAACTATGTGTGTGTTTAAAATCGACATTCGTGTTCTGTACATTTGGACCAAACGGCTCACTTAAAGAAGAGTTTATATTATTATAATTATTCTATTATTATATAAGATATATATAGAGAGACTGACGCTTGTATTTTTAAAACTGCTCATTTTTACACTTTCTACAGATGCATTTAACGGTTTTGAGGAATGAATAGGAGCACGGCAGATCTCACTGTGCTTTTGATATCGTGCCAAATGAACTTAACAAACTCAAGACCACTTTCCTTTTAGGGAACAATAACAGGGTGCCTTCACAAGCTCTCCTTTCAGTTTCACTCTTCCCAGTCCTTTTGAGGTTTAAAACGAGCCCCTGTTTTGTAAGATAATGTAGTATTTGAGTGGAATCGAAATTAAGCTGTTTTTTTAATGGCACTCATCAATTTGTATAACAAACTGTCTATGAAAAGGGAAATTAAAAAGTGGCTAAAAGCTACTGCCAGAGCTCAGTTAATCGTAATCGATGAGTTTACCAGAAGGCTTTTAAAAATTAATAATAAATACAAAAGATGTACAAGATTGTCTTCACTCACTCCACAAATAGTGACTTGATTCACTGTTTTATGTTAAAAAAGATGAAAAAACGTTTTGAATGGATATTAACATGGCCAGTTATTTATTGGTTATCTTTAACCTGACCGAATCAATGTTTGCTGTAATTCTGTCTTTCTCTCTCTTAAGGAAAATTAAATGTTTTTTTGACACCTGTAAAA

**Construction of *wnk1a-GFP***

*wnk1a*-EcoR-F: **^5’^**AATA**GAATTCTTCCACTTGGTTTAAAGCGG^3’^**

*wnk1a*-BamH1-R: **^5’^**AATA**GGATCCGCCTTCAGCAGTTCTCGATC^3’^**

(Reverse complementary: **^5’^GATCGAGAACTGCTGAAGGCGGATCC^3’^** )

Product length: 263

***wnk1a* ATG-MO:** **^5’^ACTTGACCATCTTGTCGTTGAGATT^3’^**

(reversecomplementary: **^5’^AATCTCAACGACAAGATGGTCAAGT^3’^)**

*wnk1a*-5 MM MO: **^5’^**ACTTCACGATCTTCTCCTTGACATT**^3’^**

***wnk1a* Up MO:** **^5’^TCCACCAAGTGGAGCGTGAAGTTAG^3’^**

(reversecomplementary: **^5’^CTAACTTCACGCTCCACTTGGTGGA^3’^)**

> *wnk1a-GFP*

GGGCGGTAGGCGTGTACGGTGGGAGGTCTATATAAGCAGAGCTGGTTTAGTGAACCGTCAGATCCGCTAGCGCTACCGGACTCAGATCTCGAGCTCAAGCTTC**GAATTCTTCCACTTGGTTTAAAGCGG**CCTCTTCTTGATATCAGATATTTATACTGCCACTTTTTTGCATGGATAGCATATAAGGTTCAA**CTAACTTCACGCTCCACTTGGTGGA**GAAATGTCAGAA**AATCTCAACGACAAGATGGTCAAGT**TCCTTTCCCCCCCTTCGAAGAACACCAACGGCTCCAGCTCAGACACTTTGGTGGGTGAGCATCTAGGTGTCGATGTCCGTCGTCGCCACCACACCATG**GATCGAGAACTGCTGAAGGCGGATCC**ACCGGTCGCCACC**ATGGTGAGCAAGGGCGAGGAGCTGTTCACCGGGGTGGTGCCCATCCTGGTCGAGCTGGACGGCGACGTAAACGGCCACAAGTTCAGCGTGTCCGGCGAGGGCGAGGGCGATGCCACCTACGGCAAGCTGAC**

**Construction of *wnk1b-GFP***

*wnk1b*-EcoR-F: **^5’^**AATA**GAATTCAACTCTGTGGTTCACGTGAG^3’^**

*wnk1b*-BamH1-R: **^5’^**AATA**GGATCCTGGCGTCGCTTTCTGAC^3’^**

(Reverse complementary: **^5’^GTCAGAAAGCGACGCCAGGATCC^3’^** )

Product length: 286

**zebrafish *wnk1b Up MO*** : **^5’^TGCGTAAATTTCCTGCTCTTGCTT^3’^**

(reverse complementary: **^5’^AAGCAAGAGCAGGAAATTTACGCA^3’^)**

**zebrafish *wnk1b ATG* MO: ^5’^TGGGATTTTCCGATGACATCTTTCC^3’^**

(reverse complementary: **^5’^GGAAAGATGTCATCGGAAAATCCCA^3’^)**

> *wnk1b-GFP*

GGGCGGTAGGCGTGTACGGTGGGAGGTCTATATAAGCAGAGCTGGTTTAGTGAACCGTCAGATCCGCTAGCGCTACCGGACTCAGATCTCGAGCTCAAGCTTC**GAATTCAACTCTGTGGTTCACGTGAG**CGTTTATTTTTTTCTCAATCTTTTTGTGCCACAGTTGA**AAGCAAGAGCAGGAAATTTACGCA**AAAGGTCAAAGTTTACATTTACGCTTTTTTCCAAAATTATTTATTTATACGTTCAAGTTTATTTTTCCCT**GGAAAGATGTCATCGGAAAATCCCA**ACAAGGTGGTGACCTTCTTGGCCCCGCCACCTCCAAAGAATGTGAATGGCTCCGGTTCAGACTCGCTGGTTGGTGAGAAGCTAGACACGGAG**GTCAGAAAGCGACGCCAGGATCC**ACCGGTCGCCACC**ATGGTGAGCAAGGGCGAGGAGCTGTTCACCGGGGTGGTGCCCATCCTGGTCGAGCTGGACGGCGACGTAAACGGCCACAAGTTCAGCGTGTCCGGCGAGGGCGAGGGCGATGCCACCTACGGCAAGCTGAC**
